# QTL-Seq identifies quantitative trait loci of relative electrical conductivity associated with heat tolerance in bottle gourd *(Lagenaria siceraria)*

**DOI:** 10.1371/journal.pone.0227663

**Published:** 2020-11-10

**Authors:** Hui Song, Yunping Huang, Binquan Gu

**Affiliations:** Key Lab of Cucurbit Vegetable Breeding, Ningbo Academy of Agricultural Sciences, Ningbo, Zhejiang, China; Texas Tech University, UNITED STATES

## Abstract

Heat is a major abiotic stress that seriously affects watermelon (*Citrullus lanatus*) production. However, its effects may be mitigated through grafting watermelon to heat tolerant bottle gourd (*Lagenaria siceraria*) rootstocks. Understanding the genetic basis of heat tolerance and development of reliable DNA markers to indirectly select for the trait are necessary in breeding for new varieties with heat tolerance. The objectives of this study were to investigate the inheritance of heat tolerance and identify molecular markers associated with heat tolerance in bottle gourd. A segregating F_2_ population was developed from a cross between two heat tolerant and sensitive inbred lines. The population was phenotyped for relative electrical conductivity (REC) upon high temperature treatment which was used as an indicator for heat tolerance. QTL-seq was performed to identify regions associated with heat tolerance. We found that REC-based heat tolerance in this population exhibited recessive inheritance. Seven heat-tolerant quantitative trait loci (*qHT1*.*1*, *qHT2*.*1*, *qHT2*.*2*, *qHT5*.*1*, *qHT6*.*1*, *qHT7*.*1*, and *qHT8*.*1*) were identified with *qHT2*.*1* being a promising major-effect QTL. In the *qHT2*.*1* region, we identified three non-synonymous SNPs that were potentially associated with heat tolerance. These SNPs were located in the genes that may play roles in pollen sterility, intracellular transport, and signal recognition. Association of the three SNPs with heat tolerance was verified in segregating F_2_ populations, which could be candidate markers for marker assisted selection for heat tolerance in bottle gourd. The *qHT2*.*1* region is an important finding that may be used for fine mapping and discovery of novel genes associated with heat tolerance in bottle gourd.

## Introduction

Heat stress negatively affects physiological processes, reproduction, and adaptation in crop plants, which are exacerbated by global climate change [1,2]. Watermelon, *Citrullus lanatus* var. *lanatus*, is an important vegetable crop worldwide [3]. Despite of its tropical origin, watermelon production in many parts of the world is adversely affected by high temperatures (> 35°C) during the summer months [4]. Heat tolerance is a complex trait controlled by quantitative trait loci (QTL), which makes it difficult to introgress multiple favorable alleles into recipient susceptible varieties [5]. There have been attempts to deconstruct stress tolerance into measurable components for accurate phenotyping, with the aim that QTL associated with heat tolerance may then be identified and suitable alleles may be introgressed into elite genetic backgrounds [6]. For example, cell membrane stability as an indicator of heat stress may be quantified by relative electrical conductivity (REC). REC is highly sensitive to abiotic stress [7,8] and has been used in studies of abiotic stress tolerance in a range of crops, including salinity-alkalinity tolerance in muskmelon [9], drought tolerance in *Perennial ryegrass* [10], and cold tolerance in alfalfa [11].

One method to mitigate abiotic and biotic stresses in vegetable production is grafting [12]. For example, bottle gourd, *Lagenaria siceraria* (Mol.) Standl., has been used as the rootstock for watermelon to reduce heat stress and improve performance of plant growth [13,14]. Bottle gourd is a relative of watermelon in the Cucurbitaceae family, which originated from Africa but it is now widely distributed across the tropics [15,16]. In its long-term adaptation, bottle gourd has gained excellent tolerance to high temperatures and consequently, to heat stress [17,18]. In practice, due to the close genetic relatedness between bottle gourd and watermelon, there is a high degree of grafting compatibility between the two species [19]. However, little is known about the genetic basis of heat tolerance in bottle gourd.

Understanding the genetics of heat tolerance in bottle gourd and identification of DNA markers may facilitate development of novel bottle gourd rootstocks for heat adaptation of scion through marker-assisted selection. Recent progress in genetic and genomics resources in bottle gourd is also making this possible. For example, Xu et al. [20] reported partial sequencing of the bottle gourd genome using the 454 GS-FLX Titanium sequencing platform, from which 400 SSR markers were developed. The RAD-Seq [21] technology has also been applied to an F_2_ bottle gourd population for SNP and insertion-deletions marker development [22,23]. More recently, Wu et al. [16] reported a high-quality bottle gourd genome sequence which allowed reconstruction of the most recent common Cucurbitaceae ancestor genome through comparison with available extant modern cucurbit genome resources [24–26].

In addition, recent development of high throughput sequencing and genotyping technologies is also accelerating molecular marker development and genetic mapping studies for horticulturally important traits in vegetable crops. One such technique is QTL-Seq which combines bulked-segregant analysis (BSA) and high throughput genome sequencing for quick identification of QTLs [27]. QTL-seq has been widely used in a range of crops such as sunflower [28], rice [29], sorghum [30], potato [31], and cucumber [32,33] for the efficient detection of QTLs for complex quantitative traits. QTL detection for heat tolerance has also been studied in rice [34], chickpea [35], wheat [36], and tomato [37]. In this study, we used QTL-seq to identify major loci regulating heat tolerance in bottle gourd based on REC. Putative candidate genes controlling heat tolerance and SNPs markers that are highly associated with candidate genes were identified using the available genomic sequence for bottle gourd [21].

## Materials and methods

### Plant material and phenotyping

An F_2_ population was developed from the cross between two bottle gourd inbred lines, the heat tolerant L1 (P17) and the heat stress sensitive L6 (P23) [38]. Evaluation of heat tolerance was conducted in three experiments with 147, 56, and 60 F_2_ individuals, respectively. L1, L6 and F_1_ plants (10 each) were included in each experiment. The seedlings were planted in 32-hole plastic plugs (~ 230cm^3^/hole) filled with nursery substrate (2:1 mix of turf: vermiculite) and grown in a phytotron set to 16h light/8h dark cycle (30,000lux) at 25/18°C and 80% relative humidity. The first true leaf of the seedlings was collected for DNA extraction. When the third true leaf of the seedlings began to expand, heat stress was applied by moving the seedlings into a phytotron set at 40°C temperature under 80% relative humidity and 30,000lux for 6h of continuous heat exposure.

The electrical conductivity (EC) of leaves was measured before and after heat treatment to assess cell membrane damage, as described by Zhou and Leul [39] and He et al. [40] with modifications. In brief, sampled leaves were washed using deionized water, cut into 0.5-cm pieces, and immersed in deionized water for 30 min. Then, EC of the solution was measured using a conductivity meter (PHSJ-3F, Jingmi Instruments Co., Ltd., Shanghai, China) and recorded as S1. After boiling the leaf samples for 15 min, EC of the solution at room temperature was measured again and recorded as S2. A relative EC (REC) was calculated as $\frac{\text{S}1}{\text{S}2}\times 100\%$. Leaf relative injury (LRI) was used as a metric of cell membrane damage, which was calculated as $\text{LRI}\;=\;\frac{\text{Lt}-\text{Lck}}{100-\text{Lck}}\times 100\%$, where Lt and Lck are the REC values before and after heat exposure, respectively. A larger LRI value indicates less heat tolerance [41–43].

Analysis of variance (ANOVA) and t-tests were used to determine differences in heat tolerance, as indicated by mean (±SE) LRI, among P_1_, P_2_, and F_1_ plants at *P* < 0.05 using SPSS 13.0 software [16]. For the F_2_ population, the frequency distribution of LRI was plotted as a histogram and the normal distribution was fitted using the GaussAmp function in ORIGIN 9.1 software [44].

### QTL-Seq and data analysis

Genomic DNA was isolated from young leaves of parental lines L1 and L6, and 147 F_2_ plants from the first experiment using the CTAB method [45]. For QTL-seq, two DNA pools (heat tolerance: T-pool; heat sensitive: S-pool) were constructed by mixing an equal amount of DNA from nine heat tolerant (LRI = 1 − 5%) and nine heat sensitive (LRI = 55 − 77%) individuals from the F_2_ population. Sequencing libraries of ~ 500 bp insert size of the two pools were constructed and pair-end sequenced (150 bp) at ~ 15× coverage for the two parents and ~ 20×coverage for each pool on an Illumina Hi-Seq 4000 at Shanghai Biozeron Co., Ltd. The raw sequence data were generated by Illumina base calling software CASAVA v1.8.2 [46] and raw paired end reads were trimmed and quality controlled by Trimmomatic (http://www.usadellab.org/cms/index.php?page=trimmomatic) with default setting. The high-quality clean reads from L1, L6, T-pool and S-pool were aligned against the reference genome of bottle gourd cv. Hangzhou Gourd (http://cucurbitgenomics.org/organism/13, [21]) using BWA software (http://bio-bwa.sourceforge.net/, [47]). After removing the duplicate reads using Picard Tools (http://picard.sourceforge.net/), SNPs were detected from the valid BAM file using the GATK “UnifiedGenotyper” function (http://www.broadinstitute.org/gatk/). Low-quality SNPs with quality value < 20 and read depth coverage < 4× or > 200× were excluded [48]. SNPs with the consistent differential base type with two parents were remained.

The SNP-index for each SNP position was calculated for the T-pool and S-pool using the formula:

$$\text{SNP}-\text{index}\;(\text{at}\;\text{a}\;\text{position})\;=\frac{\text{count}\;\text{of}\;\text{alternated}\;\text{base}}{\text{count}\;\text{of}\;\text{reads}\;\text{aligned}}$$

[49]. To identify candidate regions for heat tolerance QTLs, the Δ(SNP-index) for all the SNP positions with given read depths under the null hypothesis of no QTLs was obtained by subtracting the SNP-index of T-pool from the S-pool [32]. The statistical confidence intervals of Δ(SNP-index) were plotted [27]. For each read depth, 95% and 99% confidence intervals of Δ(SNP-index) were obtained following Takagi et al. [27]. The regions (*P* < 0.01) were then designated as QTLs.

To identify the parent that contributed to a putative QTL, the profile of L1 allele frequency difference (L1AFD) between the T-pool and S-pool was plotted using a 1 Mbp sliding window moving across the genome with a fixed step length of smoothing window size of 10 kb. The L1 allele frequency within each window in each pool was estimated using the formula developed by Yang et al. [50]. For a putative QTL, a positive value for L1AFD indicated that L1 increased heat tolerance and a negative value indicated that L1 decreased heat tolerance [33,50].

For the polymorphic SNPs located in the genomic regions that harbored the major-effect heat tolerance QTL, further functional annotation was completed using the available bottle gourd genome data (http://cucurbitgenomics.org/organism/13) and Uniprot database (www.uniprot.org), using ANNOVAR analysis (http://www.openbioinformatics.org/annovar/) to detect putative candidate genes [21].

### SNP marker development and selection

Based on gene annotations and putative functions, five nonsynonymous candidate SNPs located in the *qHT2*.*1* interval that are related to heat stress tolerance were selected for marker development. Flanking sequences were used for PCR primer development using Primer 6.0 soft (http://www.PromerBiosoft.com). The five SNPs were genotyped for four heat tolerant and four sensitive F_2_ individuals which were used to generate the two DNA pools. If the SNPs showed polymorphism between the heat tolerant and sensitive lines, they were used to genotype additional plants. Eighteen new individuals including six heat tolerant individuals (LRI < 10%) and twelve sensitive individuals (LRI > 10%) were selected from the same F_2_ population used for DNA pool construction. The LRI threshold was set to 10% because the mean LRI value of the tolerance parent L1 was 9.88±5.00%. PCR and Sanger sequencing were used and sequence results were visualized and checked using the SnapGene software. We used trait-marker association to select the most promising SNP markers.

## Results

### LRI as an indicator of heat tolerance

**Fig 1:**
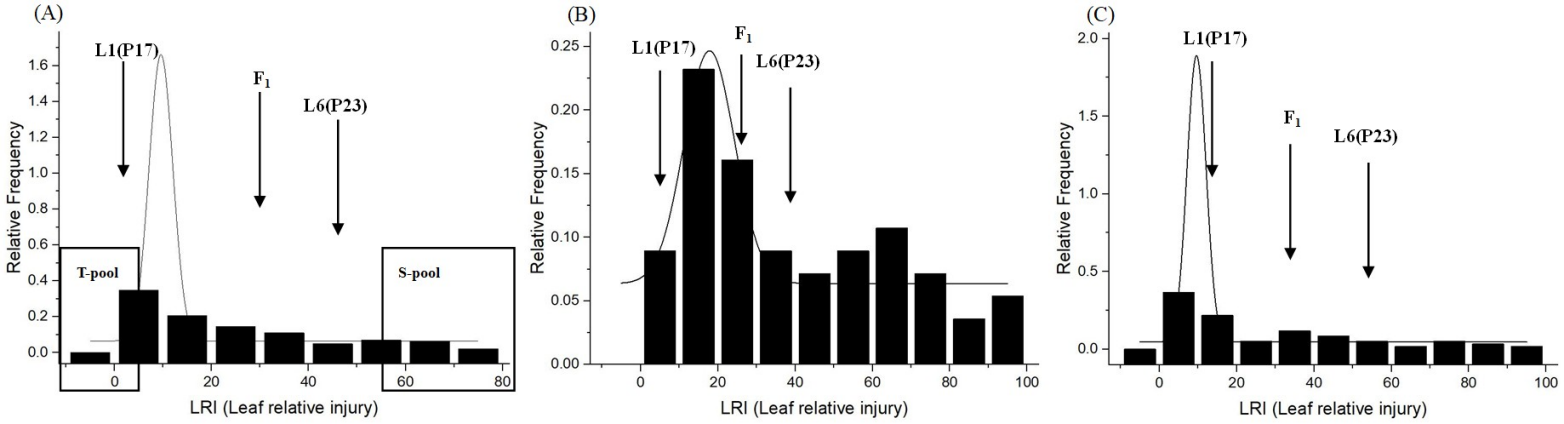
Frequency distribution of leaf relative injury (LRI) among three F_2_ populations with 147 individuals (A), 56 individuals (B), and 60 individuals (C). L1: heat tolerant parent P17; L6: heat sensitive parent P23. Distribution of L1 near the origin of the x-axis indicates negative transgressive segregants, while distribution of L6 indicates positive transgressive segregants under heat-stress conditions. DNA of eighteen seedlings was selected from an F_2_ population with 147 individuals **(A)** with extreme phenotypes (low and high LRI values) to develop tolerant and sensitive pools.

There were significant differences in the mean LRI values between L1 (P17: 9.88±5.00%) and L6 (P23: 47.26±10.78%) under heat stress conditions (Table 1). The mean LRI value of F_1_ (31.98±6.64%) was significantly higher than that of L1 but not significantly different with that of L6, suggesting the recessive nature of heat tolerance. The LRI of the F_2_ progeny ranged from 0.34% to 97.49%, with mean LRI value of 29.68±8.52% (Table 1). Transgressive segregation was observed on both sides of the distribution. Overall, the distributions of the three F_2_ populations were slightly skewed towards L1 (P17) and showed Gaussian segregation (Fig 1A–1C).

**Table 1:** Mean leaf relative injury (LRI) values of parental lines, F_1_ and three F_2_ populations under heat stress conditions.

Type	Code	LRI		Reaction
		Mean±SE	Range	
Tolerance parent	L1 (P17)	9.88±5.00a*	4.28 − 20.25	Tolerant
Sensitive parent	L6 (P23)	47.26±10.78b	33.52 − 59.32	Sensitive
Mid-parent value	-	28.32±7.89	-	-
F_1_ population	L1 × L6	31.98±6.64b	19.95 − 43.95	Sensitive
F_2_ population	-	29.68±8.52	0.34 − 97.49	-

*P < 0.05.

### Sequence data

High throughput Illumina sequencing yielded 31.9, 32.7, 40.9, and 40.9 M 150-bp paired-end raw reads from L1, L6, T-pool, and S-pool, respectively. After trimming and filtering, more than 80% of reads were mapped to the reference genome of bottle gourd cv. Hangzhou Gourd (313.4 Mbp, Table 2). Specifically, 23.0, 24.4, 29.0, and 29.5 million short reads were mapped for L1 (10.10×depth coverage or 92.75% coverage), L6 (10.85×depth coverage or 92.85% coverage), T-pool (12.71×depth coverage or 93.25% coverage), and S-pool (12.92×depth coverage or 93.25% coverage), respectively. Lower sequencing depth (about 10×) and higher coverage (about 92%) of L1 and L6 showed the close genetic relationship between the parental lines and cv. Hangzhou Gourd. In our study, the high-quality bottle gourd Hangzhou Gourd genome sequence [21] was used to map each DNA pool. The sequence data of parental lines were used to verify SNPs detected from two pools. Thus, SNPs between the two pools with alleles not inherited from either parent were filtered out.

**Table 2:** Main statistics of resequencing and SNP calling in two parental lines and two pools.

Sample	Number of raw reads	Trimmed and filtered reads		Uniquely mapped reads		Average Depth	Coverage (%)^a^	Number of SNPs		
		Number	%	Number	%			Total	Homozygous	Heterozygous
L1	31,863,484	28,368,754	89.03	22,974,995	80.99	10.10	92.75	476,940	345,242	131,698
L6	32,686,118	29,026,164	88.80	24,419,983	84.13	10.85	92.85	513,552	418,749	94,803
T-pool	40,647,374	35,340,578	86.94	29,026,863	82.13	12.71	93.25	543,798	359,267	184,531
S-pool	40,930,720	35,936,306	87.80	29,457,284	81.97	12.92	93.25	549,415	361,111	188,304

^a^ size of the bottle gourd reference genome is 313,397,697 bp (Xu et al. 2014 [22]).

Based on the uniquely mapped reads, 543,798 and 549,415 SNP were identified from the T-pool and S-pool, respectively. Among them, 359,267 and 361,111 were homozygous in T-pool and S-pool; 184,531 and 188,304 SNPs were heterozygous in the both pools, respectively (Table 2). After filtering our low quality SNPs, 153808 SNPs between the T-pool and S-pool were kept for further analysis.

### Identification of QTL for heat tolerance from QTL-Seq

**Fig 2:**
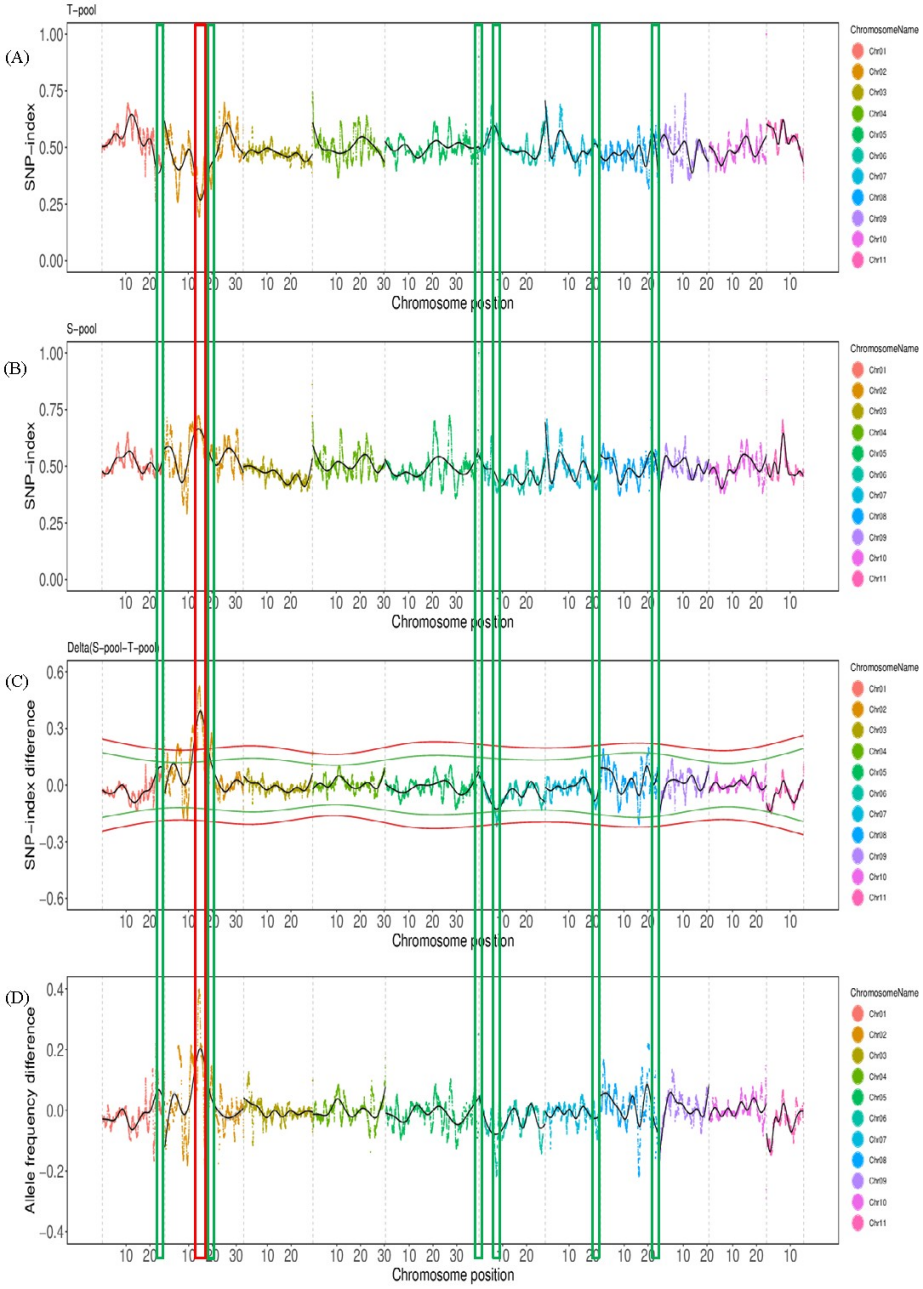
SNP-index plots of T-pool (A) and S-pool (B) and Δ(SNP-index) plot (C) from the QTL-Seq analysis. The x-axis represents the position of eleven chromosomes and the y-axis represents SNP-index or Δ(SNP-index) value. The Δ(SNP-index) plot **(C)** shows statistical confidence intervals under the null hypothesis of no QTL (*P* < 0.01). The red and green wavy line means 99% and 95% confidence intervals, respectively. The promising genomic region identified for LRI associated with heat tolerance is highlighted at 11.03–19.25 Mb on Chromosome 2 by red frame. The other six QTL region with high Δ(SNP-index) value are highlighted by green frame. **(D)** Profile of the tolerant parent (L1) allele frequency difference (L1AFD).

The SNP-index graphs were generated for the T-pool (Fig 2A) and S-pool (Fig 2B). The Δ(SNP-index) was determined by subtracting the SNP-index of T-pool from the S-pool and plotted against the genome positions (Fig 2C). By examining the Δ(SNP-index) plot, we identified seven genomic regions that exhibited high Δ(SNP-index) values (Table 3) on chromosomes 1, 2, 5, 6, 7 and 8. These were candidate regions harboring heat tolerance QTLs (Fig 2C). Among them, the peak on Chr 5 was the highest, followed by the peaks on Chr 2 (*qHT2*.*1*), Chr 1, Chr 7, Chr 6, Chr 8 and Chr 2 (*qHT2*.*2*). The two adjacent peaks on Chr 2 contained most of the SNPs, 9052 and 1355, respectively. The peak region on Chr 5 only contained two SNPs (Table 3). The QTL associated with these regions were designated as *qHT1*.*1*, *qHT2*.*1*, *qHT2*.*2*, *qHT6*.*1*, *qHT7*.*1*, and *qHT8*.*1*, respectively, hereinafter.

**Table 3:** QTLs identified from QTL-Seq that conferred heat tolerance in bottle gourd.

Region	QTL	SNP Number	SNP-index		Δ(SNP-index)	P-value	Interval	L1AFD
			T-pool	S-pool				
Chr01:26,260,000–27,279,999	*qHT1*.*1*	12	0.3713	0.622	0.25	0.000413811	1,019,999	0.13
Chr02:11,030,000–19,249,999	*qHT2*.*1*	9052	0.3092	0.6285	0.32	9.79E-06	8,219,999	0.24
Chr02:19,410,000–20,989,999	*qHT2*.*2*	1355	0.3579	0.5463	0.19	0.006172464	1,579,999	0.11
Chr05:39,390,000–40,419,999	*qHT5*.*1*	2	0.45	0.8333	0.38	1.42E-07	1,029,999	0.25
Chr06:7,460,000–8,459,999	*qHT6*.*1*	934	0.5917	0.379	-0.21	0.001783751	999,999	-0.21
Chr07:23,220,000–24,229,999	*qHT7*.*1*	5	0.3648	0.5812	0.22	0.001986976	1,009,999	0.23
Chr08:24,880,000–25,889,999	*qHT8*.*1*	4	0.1853	0.3988	0.21	0.002248176	1,009,999	0.19

The L1AFD value of a QTL reflects the magnitude and direction of L1 allele effect of the associated QTL. Thus, *qHT5*.*1* seemed to have the largest effect on REC, followed by *qHT2*.*1*, *qHT7*.*1*, *qHT6*.*1*, *qHT8*.*1*, *qHT1*.*1* and *qHT2*.*2*. The tolerant alleles of *qHT5*.*1*, *qHT2*.*1*, *qHT6*.*1*, *qHT8*.*1*, *qHT1*.*1* and *qHT2*.*2* were derived from the tolerant parent L1 (L1AFD value > 0) while the *qHT7*.*1* (L1AFD value < 0) was from the susceptible parent L6 (Fig 2D and Table 3).

Although *qHT5*.*1* showed the highest values of Δ(SNP-index) and L1AFD, only two SNPs were associated with this QTL, both of which were located in intergenic regions. Thus, *qHT2*.*1*, with Δ(SNP-index) value of 0.32 (*P* < 0.01), L1AFD value of 0.24, and containing 9052 SNPs, was selected as a promising major QTL controlling heat tolerance.

### Identification of heat tolerance candidate genes in *qHT2*.*1* interval

In general, the confidence intervals of the QTL regions based on Δ(SNP-index) peaks were large, with a maximum of 8.22 Mbp for *qHT2*.*1* (Table 3). There were 9052 SNPs between T-pool and S-pool in this region, of which 527 were in genic regions. Regions harboring the 279 SNPs were annotated by BLASTx against the non-redundant protein database (S1 Table, [51]), which identified 62 non-synonymous SNPs with Δ(SNP-index) ≥ 0.5 (S2 Table). Gene ontology classification revealed these 62 genes were mainly associated with biological processes (S1 Fig), such as metabolism; cellular components, such as the cell membrane; and molecular function, such as catalytic activity. The biological processes of the 62 candidate genes were retrieved from the available bottle gourd genome data and Uniprot database. Thirty four of the 62 genes seemed to be related with heat stress and are involved in biological processes, pollen and flower sterility, oxidative stress response, autophagy, and abiotic stress response (S3 Table).

### Development of SNP markers associated with heat tolerance

Among the 34 candidate genes potentially associated with tolerance, five genes harboring five nonsynonymous SNPs were selected for primer design (Table 4). Among them, SNP 2 (BG_GLEAN_10022642, annexin 5) was related to pollen and flower sterility. SNP 15 (BG_GLEAN_10022339, PQL1 and PQL2) and SNP 16 (BG_GLEAN_10022589, serine/threonine-protein kinase) were related to signal recognition. SNP 26 (BG_GLEAN_10022734, MuDR) and SNP 31 (BG_GLEAN_10022727, AP-3 adaptor complex) were related to intracellular transport. All these five SNPs altered the predicted protein sequences of the genes (Table 4).

**Table 4:** Primer design, position of SNPs located in their respective gene and the protein sequence alteration.

SNP	Gene	Mutation	Chr	Pos	Protein	Primer-F	Tm-R	Primer-R	Tm-F
15	BG_GLEAN_10022339	C->T	2	12878537	V → I	TACTCCTCGGCGTCTAACCA	60.0	TGAGAAGCAGCCTCTCGTTG	60.0
26	BG_GLEAN_10022734	A->G	2	17634545	T → A	AGGTAGGCCCGAAGACTCAT	60.0	GGGAGGGCAGCTTGTTACTT	60.0
2	BG_GLEAN_10022642	C->A	2	16355258	S → Y	CGCGCTATGAAGGTCCAGAA	60.2	CCCAGGATTCTCAGCACACA	60.0
16	BG_GLEAN_10022589	A->G	2	15709303	K → E	GAGCCCAGTTGGTTCCTTGA	60.0	CATCTGCGATCTCCGTCCTG	60.0
31	BG_GLEAN_10022727	T->A	2	17457363	C → S	CGTCTATGGGGGTTGGCAAT	60.1	CGAGAGTCCTGCAGCAGAAA	60.0

Genotyping of 10 heat tolerant and 16 sensitive F_2_ individuals (S2 and S3 Figs, Table 5) showed two of the five SNPs (SNP 15 and SNP 26) had no polymorphism between the heat tolerant and sensitivity lines. SNP 2 (CC), SNP 16 (GG) and SNP 31 (TT) were homozygous in heat tolerant individuals, which were with homozygous (AA, AA, AA) or heterozygous genotype (AC, AG, AT) in heat sensitive plants, respectively. The alleles of the three SNPs (SNP 2, SNP 16 and SNP 31) showed good correlations with heat tolerance in the two parents, the two pools and tested F_2_ plants (Table 5) suggesting their potential value as makers for marker-assisted selection of heat tolerance.

**Table 5:** Sanger sequencing verification of five candidate SNP loci in 10 heat tolerant and 16 sensitive F_2_ individuals.

SNP	Gene	L1	L6	Heat tolerant individuals										Heat sensitive individuals															
				1	2	3	4	5	6	7	8	9	10	1	2	3	4	5	6	7	8	9	10	11	12	13	14	15	16
15	BG_GLEAN_10022339	T	C	C	C	C	C	-*	-	-	-	-	-	C	C	C	C	-	-	-	-	-	-	-	-	-	-	-	-
26	BG_GLEAN_10022734	G	A	AG	AG	AG	AG	-	-	-	-	-	-	AG	AG	AG	AG	-	-	-	-	-	-	-	-	-	-	-	-
2	BG_GLEAN_10022642	C	A	C	C	C	C	C	C	C	C	C	C	A	AC	AC	A	A	A	AC	A	AC	AC	AC	AC	A	AC	AC	AC
16	BG_GLEAN_10022589	G	A	G	G	G	G	G	G	G	G	G	G	A	A	A	A	A	A	AG	A	AG	AG	AG	AG	A	AG	AG	AG
31	BG_GLEAN_10022727	T	A	T	T	T	T	T	T	T	T	T	T	A	AT	AT	A	A	A	AT	A	AT	AT	AT	AT	A	AT	AT	AT

*SNP 15 and SNP 26 had no difference between the four heat tolerant lines and four heat sensitivity lines, so they were not genotyped for the 18 lines additionally.

## Discussion

### Parental lines were not used as reference genome

Based on QTL-seq protocol [27], the genome sequence of either of the two parents can be used as a reference to map the DNA pool [32,52]. This would make it easier to identify the source of the SNPs and to tell from which parent the allele responsible for the QTL region came from. But since there is a close genetic relationship between the parental lines used in this study and cv. Hangzhou Gourd, the cultivar used to develop the high-quality reference genome for bottle gourd [19], the available genome sequence for the latter was used as reference in our study. The sequence data of parental lines were mainly applied to ensure the differential SNPs detected from two pools were inherited from either parent.

Moreover, the L1 allele frequency difference (L1AFD) was plotted to identify the size of the effect and direction of action of the parental allele for each QTL [50]. The profile of L1AFD indicated that except for *qHT7*.*1* (L1AFD < 0) derived from the susceptible parent L6, the other six tolerant alleles of *qHT5*.*1*, *qHT2*.*1*, *qHT6*.*1*, *qHT8*.*1*, *qHT1*.*1* and *qHT2*.*2* (L1AFD value > 0) were all from the tolerant parent L1.

The *qHT2*.*1* had higher values of Δ(SNP-index) and L1AFD, and contained the largest number of detected SNPs (9052). Thus, *qHT2*.*1* was identified as the most promising major-effect QTL for heat tolerance. For cucumber [33], credible QTLs associated with downy mildew resistance were detected via QTL-Seq method and double checked by conventional method. In their research, the reads of two DNA pools were mapped against the reference genome of cucumber cv. Chinese Long, not mapped against the parental line of TH118FLM or WME. The genetic relationship among cucumber was narrow. The polymorphism information content value of SSR and SCAR was only 0.65 [53]. For sunflower [54], the reference genome for *Helianthus annuus* was used and not the parental lines 902R or 906R, for the identification of candidate resistance gene to broomrape by BSA-seq.

### Genetics basis of heat tolerance in bottle gourd

Plants have evolved a range of metabolic responses, such as antioxidant activity, membrane lipid unsaturation, protein stability, gene expression and translation, and accumulation of compatible solutes [55], to cope with heat stress through the activation of stress-response genes [56]. Heat tolerance, which could be characterized by different phenotypic and physiological parameters, has been shown to be a quantitative trait [57]. The quantitative nature of heat tolerance has been revealed in a number of crop plants, like wheat [58,59], rice [34], and broccoli [60,61]. However, there are also reports of monogenic and oligogenic responses to heat tolerance [62,63]. Little is known about the inheritance of heat tolerance in bottle gourd. In this study, the F_1_ of the L1×L6 was heat sensitive suggesting that heat tolerance is a recessive trait in bottle gourd. We also identified major-effect QTL for this trait. Thus, our work represented the first to report REC related to heat tolerance in this important vegetable crop.

In the present study, *qHT2*.*1* was identified as the most promising major QTL for heat tolerance. Similar results have been reported for African rice (*Oryza glaberrima* Steud.) [64]. It was reported that a major QTL that controls the survival rate of seedlings exposed to high temperatures was governed by the *Thermo tolerance 1* gene. The gene encodes a 23S proteasome subunit that degrades ubiquitinated cytotoxic denatured proteins formed due to high temperature stress. Major-effect QTL control of complex traits, such as heat tolerance, may be explained by inheritance patterns of the trait [13] and action of major regulator genes that may switch off subordinate genes, if a key gene is mutated [65].

### Annotation of heat tolerance candidate genes

Functional genomics studies in plants facilitate the elucidation of candidate genes and their relationship with traits [35]. For example, heat stress affects pollen and flower sterility [66]. Of the 34 candidate genes, four were found to have key roles in gametogenesis [67], pollen development [68,69], or Ogura cytoplasmic male sterility [70]. Heat-stress also causes cellular damage by oxidative stress and toxicity due to reactive oxygen species (ROS) formation [71,72]. Antioxidants scavenge ROS to mitigate oxidative stress. We found four putative candidate genes that had a role in defying oxidative stress and recovering plants from heat-stress damage [73–76]. Many reports have mentioned transcription factors [77], binding factors [78,79], intracellular transporters [80,81], and enzymes [82,83] that are responsible for abiotic tolerance in different crops. The signaling molecules [84] and autophagy [85] also play important roles in plant responses to stress.

### Markers and candidate genes associated with heat tolerance in bottle gourd

In this study, three SNPs with contrasting functional annotation were found to be associated with heat tolerance in bottle gourd. The three SNPs were located in three genes including those encoding for homologs of annexin 5 (BG_GLEAN_10022642, SNP 2), AP-3 adaptor complex protein (BG_GLEAN_10022727, SNP 31), and serine/threonine-protein kinase (BG_GLEAN_10022589, SNP 16). A common response of crop plants to temperature stress is significant yield loss, as a result of effects on spikelet fertility in rice [86], pod set in lentil [65], and pollen sterility in canola [66]. Annexins are members of the ubiquitous family of proteins present in eukaryotic organisms [87] and localized in various subcellular compartments [88]. They are known to be involved in a variety of cellular processes, such as maintenance of vesicular trafficking, cellular redox homeostasis, actin binding, and ion transport [89], due to their calcium- and membrane-binding capacity. Annexin 5 is involved in pollen grain development and germination, and pollen tube growth through the promotion of calcium-modulated endo-membrane trafficking [90]. For example, down-regulation of Arabidopsis annexin 5 in transgenic Ann5-RNAi lines has been shown to cause sterility in pollen grains [68].

Adaptor proteins that are involved in protein trafficking and sorting [91–93] may recognize cargo and coat proteins during vesicle formation [94]. AP-3 was first identified in mammalian cells and probably functions as a clathrin adaptor [95]. Losses in AP-3 function reduce seed germination potential [96,97], due to mistargeted protein S-ACYL transferase10 that is critical for pollen tube growth during dynamic vacuolar organization in Arabidopsis [98].

Serine/threonine-protein kinases (STKs) are involved in signal transduction networks to coordinate growth and differentiation of cell responses to extracellular stimuli [99]. In a range of smut pathogenesis-related biological processes, Huang et al. [84] reported that STKs may act as receptors or signaling factors, such as in Ca^2+^ signaling, that then activate defense responses. Umezawa et al. [100] reported that SRK2C is annotated as a putative and osmotic-stress-activated STK in Arabidopsis. It might mediate signal transduction and regulate a series of drought stress-response genes that enhance expression of 35S:SRK2C-GFP to improve drought tolerance in plants.

## Conclusions

Understanding the genetic basis of heat tolerance and development of reliable DNA markers to indirectly select for the trait are important in breeding for new varieties with heat tolerance. In this study, we show that REC could be used as an indicator for heat tolerance, which exhibited recessive inheritance. We also identified three SNPs that are associated with heat tolerance. The three SNPs were identified in genes regulating pollen sterility, intracellular transport, and signal recognition. These SNPs can be used in marker-assisted selection for heat tolerance in bottle gourd. Intensity of global warming will increase over the next two to three decades and will exacerbate challenges for the cultivation of Cucurbitaceae plants. Identification of heat tolerant genotypes of bottle gourds that can be used to improve heat tolerance of other Cucurbitaceae plants, such as watermelon, through grafting techniques may mitigate these challenges. These results revealed the novel region of 11.03 − 19.25 Mb on Chr 2 harboring *qHT2*.*1* that may provide the basis for further exploration and fine mapping of novel genes associated with heat tolerance in bottle gourd.

## Supporting information

S1 Fig GO results for 62 candidate genes located in the *qHT2*.*1* region. (TIF)

S2 Fig Genotyping of four tolerant and four sensitive F_2_ individuals derived from five candidate SNPs. Eight individuals were the member of tow DNA pools including four heat tolerant F_2_ (**A**, **C**, **E**, **G**, **I**) and four heat sensitive F_2_ (**B**, **D**, **F**, **H**, **J**). **(A**, **B)** SNP15: BG_GLEAN_10022339. **(C**, **D)** SNP26: BG_GLEAN_10022734. **(E**, **F)** SNP 2: BG_GLEAN_10022642. **(G**, **H)** SNP 16:BG_GLEAN_10022589. **(I**, **J)** SNP 31: BG_GLEAN_10022727. The SNP loci are shaded and indicated with a red arrow.
(TIF)

S3 Fig Genotyping of six tolerant and twelve sensitive F_2_ individuals derived from three polymorphic SNPs. Eighteen individuals were the additional new lines, including six heat tolerant F_2_ (**A**, **C**, **E**) and twelve sensitive F_2_ (**B**, **D**, **F**). **(A**, **B)** SNP 2: BG_GLEAN_10022642. **(C**, **D)** SNP 16:BG_GLEAN_10022589. **(E**, **F)** SNP 31: BG_GLEAN_10022727. The SNP loci are shaded and indicated with a red arrow.
(TIF)

S1 Table Predicted and annotated SNPs in the *qHT2*.*1* region. (DOCX)

S2 Table Details of 62 nonsynonymous and stoploss type of SNPs in the *qHT2*.*1* region. (DOCX)

S3 Table Function annotation of candidate genes. (DOCX)
