# Supplementary figures and images for: QTL-Seq identifies quantitative trait loci of relative electrical conductivity associated with heat tolerance in bottle gourd (Lagenaria siceraria)
Source: PLoS One. 2020 Nov 10;15(11):e0227663. doi: 10.1371/journal.pone.0227663 (PMC7654804; doi:10.1371/journal.pone.0227663)

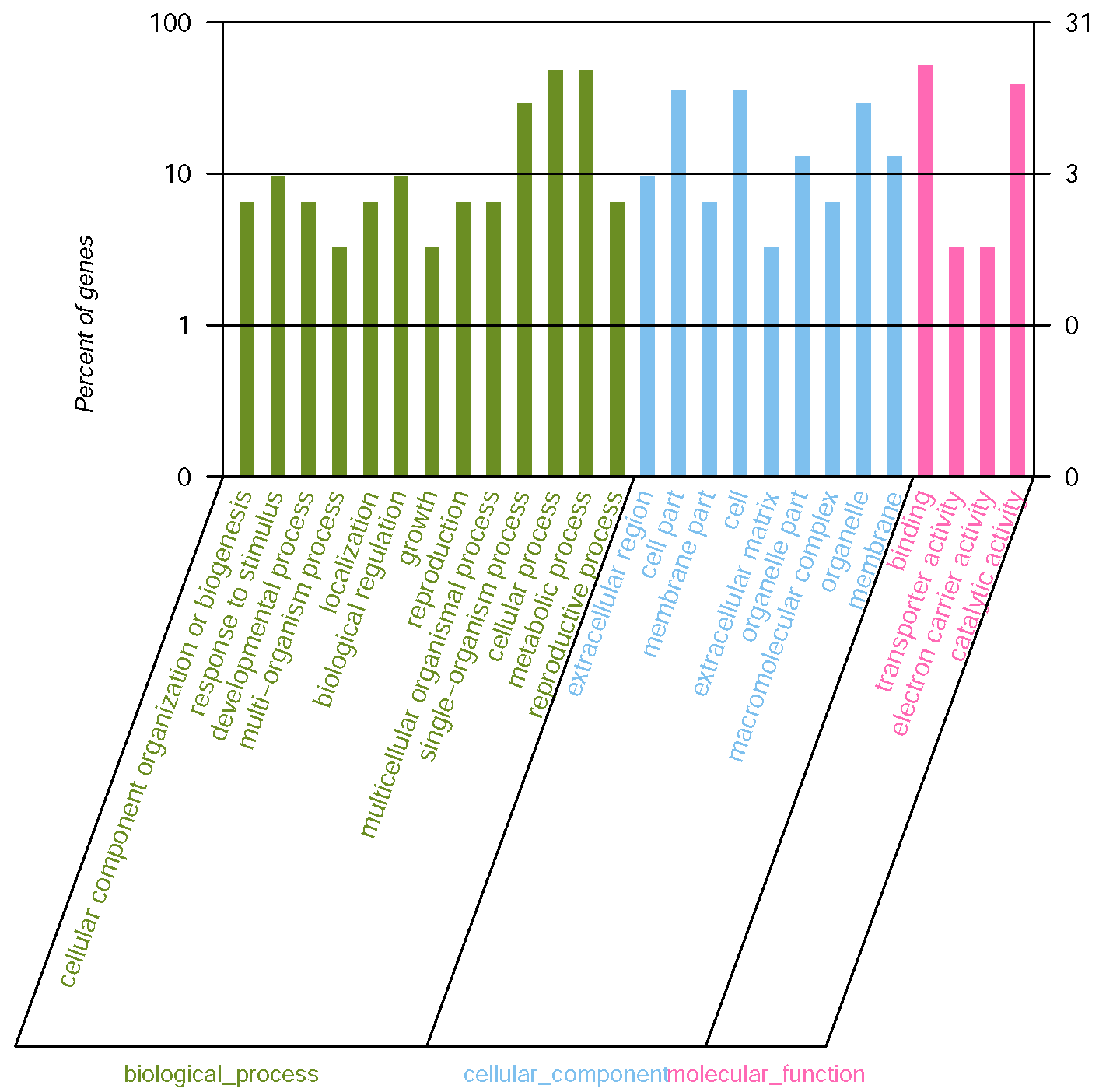

Supplement: S1 Fig — (TIF) [file pone.0227663.s001.tif]

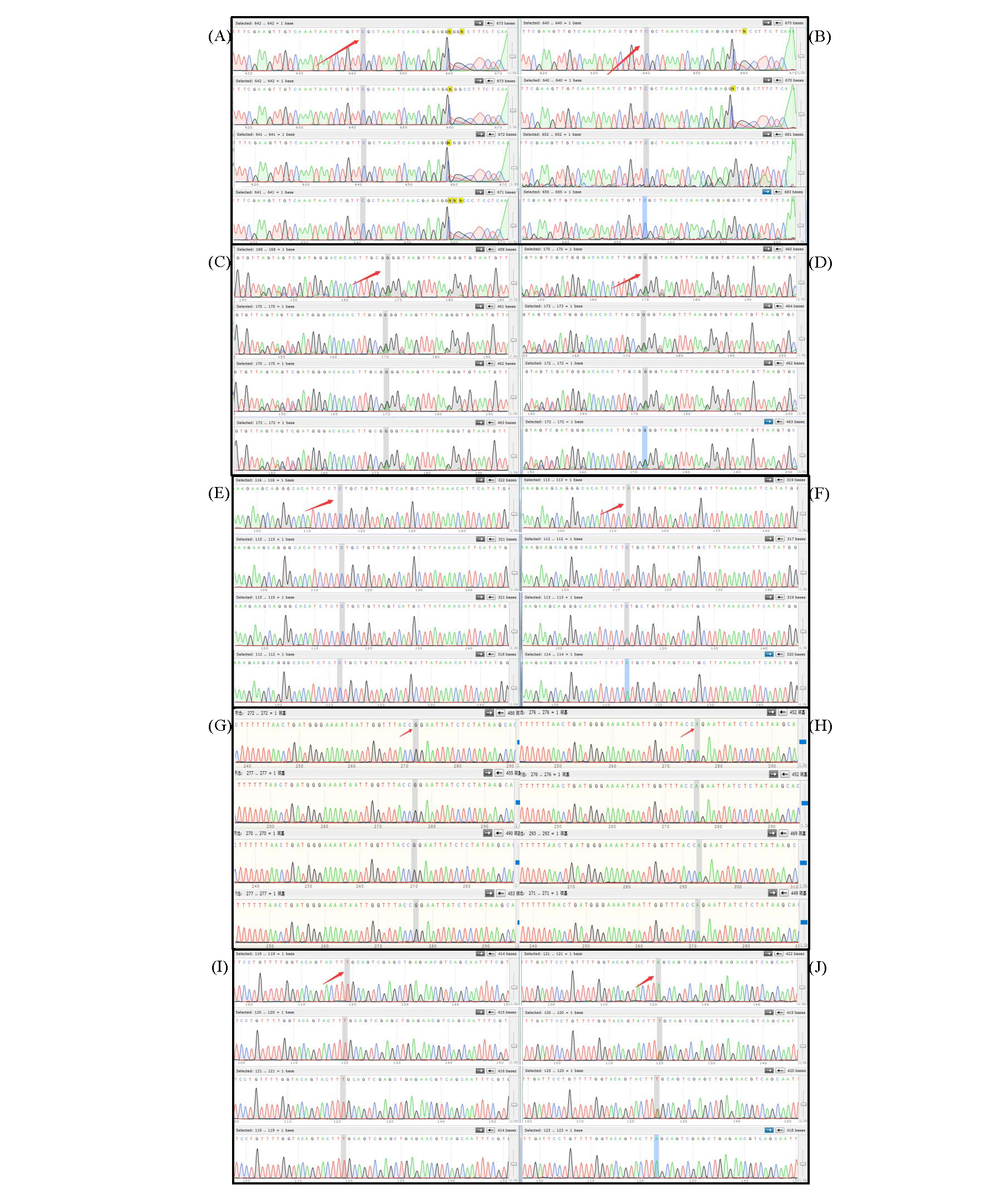

Supplement: S2 Fig — Eight individuals were the member of tow DNA pools including four heat tolerant F2 (A, C, E, G, I) and four heat sensitive F2 (B, D, F, H, J). (A, B) SNP15: BG_GLEAN_10022339. (C, D) SNP26: BG_GLEAN_10022734. (E, F) SNP 2: BG_GLEAN_10022642. (G, H) SNP 16:BG_GLEAN_10022589. (I, J) SNP 31: BG_GLEAN_10022727. The SNP loci are shaded and indicated with a red arrow. (TIF) [file pone.0227663.s002.tif]

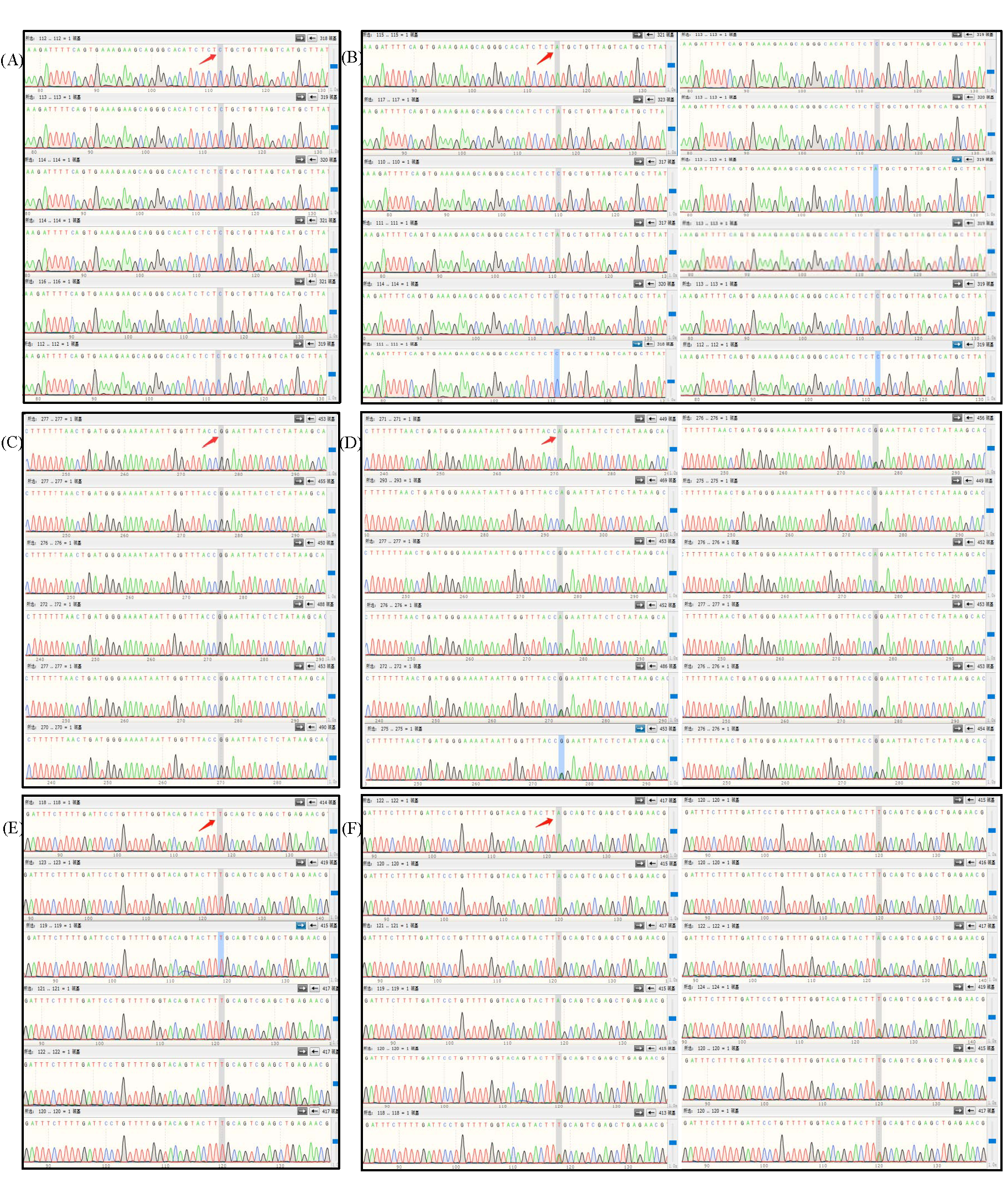

Supplement: S3 Fig — Eighteen individuals were the additional new lines, including six heat tolerant F2 (A, C, E) and twelve sensitive F2 (B, D, F). (A, B) SNP 2: BG_GLEAN_10022642. (C, D) SNP 16:BG_GLEAN_10022589. (E, F) SNP 31: BG_GLEAN_10022727. The SNP loci are shaded and indicated with a red arrow. (TIF) [file pone.0227663.s003.tif]
