# Supplementary material for: QTL-Seq identifies quantitative trait loci of relative electrical conductivity associated with heat tolerance in bottle gourd (Lagenaria siceraria)
Source: PLoS One. 2020 Nov 10;15(11):e0227663. doi: 10.1371/journal.pone.0227663 (PMC7654804; doi:10.1371/journal.pone.0227663)
Supplement: S1 Table — (DOCX) [file pone.0227663.s004.docx]

**S1 TABLE** Predicted and annotated SNPs in the *qHT2.1* region

| Chr | Gene | function |
| --- | --- | --- |
| Chr02:11030000-19249999 | BG_GLEAN_10022205 | \| Symbols: \| FUNCTIONS IN: molecular_function unknown; INVOLVED IN: biological_process unknown; LOCATED IN: cellular_component unknown; EXPRESSED IN: 22 plant structures; EXPRESSED DURING: 14 growth stages; CONTAINS InterPro DOMAIN/s: Uncharacterised conserved protein UCP017207, transmembrane protein 85 (InterPro:IPR016687), Protein of unknown function DUF1077 (InterPro:IPR009445); Has 395 Blast hits to 395 proteins in 185 species: Archae - 0; Bacteria - 0; Metazoa - 139; Fungi - 122; Plants - 51; Viruses - 0; Other Eukaryotes - 83 (source: NCBI BLink). \| chr5:3408422-3409784 FORWARD LENGTH=187 |
| Chr02:11030000-19249999 | BG_GLEAN_10022206 | \| Symbols: UBP22 \| ubiquitin-specific protease 22 \| chr5:3410638-3412559 FORWARD LENGTH=557 |
| Chr02:11030000-19249999 | BG_GLEAN_10022207 | \| Symbols: ATGPX1, GPX1 \| glutathione peroxidase 1 \| chr2:10668134-10669828 FORWARD LENGTH=236 |
| Chr02:11030000-19249999 | BG_GLEAN_10022208 | \| Symbols: eIFiso4G1 \| MIF4G domain-containing protein / MA3 domain-containing protein \| chr5:23439755-23443433 FORWARD LENGTH=776 |
| Chr02:11030000-19249999 | BG_GLEAN_10022209 | \| Symbols: \| Protein phosphatase 2C family protein \| chr4:15406685-15408589 REVERSE LENGTH=357 |
| Chr02:11030000-19249999 | BG_GLEAN_10022210 | \| Symbols: PGR3 \| proton gradient regulation 3 \| chr4:15403020-15406358 FORWARD LENGTH=1112 |
| Chr02:11030000-19249999 | BG_GLEAN_10022211 | \| Symbols: \| Sas10/Utp3/C1D family \| chr5:8643782-8646306 FORWARD LENGTH=217 |
| Chr02:11030000-19249999 | BG_GLEAN_10022212 | \| Symbols: \| unknown protein; Has 42 Blast hits to 42 proteins in 10 species: Archae - 0; Bacteria - 0; Metazoa - 0; Fungi - 0; Plants - 42; Viruses - 0; Other Eukaryotes - 0 (source: NCBI BLink). \| chr4:15400803-15401105 REVERSE LENGTH=100 |
| Chr02:11030000-19249999 | BG_GLEAN_10022213 | \| Symbols: \| Endomembrane protein 70 protein family \| chr5:3424910-3427797 REVERSE LENGTH=648 |
| Chr02:11030000-19249999 | BG_GLEAN_10022215 | \| Symbols: \| Endomembrane protein 70 protein family \| chr5:3424910-3427797 REVERSE LENGTH=648 |
| Chr02:11030000-19249999 | BG_GLEAN_10022216 | _ |
| Chr02:11030000-19249999 | BG_GLEAN_10022217 | \| Symbols: \| ATP-dependent caseinolytic (Clp) protease/crotonase family protein \| chr4:15387365-15390290 REVERSE LENGTH=409 |
| Chr02:11030000-19249999 | BG_GLEAN_10022218 | \| Symbols: NRPD2A \| nuclear RNA polymerase D2A \| chr3:8567971-8573819 REVERSE LENGTH=1172 |
| Chr02:11030000-19249999 | BG_GLEAN_10022219 | \| Symbols: ATCM2, CM2 \| chorismate mutase 2 \| chr5:3430691-3432272 REVERSE LENGTH=265 |
| Chr02:11030000-19249999 | BG_GLEAN_10022220 | \| Symbols: EIF-5A, ELF5A-1, ATELF5A-1, EIF5A \| eukaryotic elongation factor 5A-1 \| chr1:4773631-4774668 FORWARD LENGTH=158 |
| Chr02:11030000-19249999 | BG_GLEAN_10022221 | \| Symbols: \| WRKY family transcription factor \| chr4:15385454-15386775 FORWARD LENGTH=344 |
| Chr02:11030000-19249999 | BG_GLEAN_10022222 | \| Symbols: \| Protein of unknown function (DUF616) \| chr1:19764567-19766870 REVERSE LENGTH=540 |
| Chr02:11030000-19249999 | BG_GLEAN_10022223 | \| Symbols: \| Ubiquitin-conjugating enzyme family protein \| chr1:19757072-19759474 REVERSE LENGTH=543 |
| Chr02:11030000-19249999 | BG_GLEAN_10022224 | \| Symbols: \| Nucleic acid-binding, OB-fold-like protein \| chr4:6818162-6818806 FORWARD LENGTH=141 |
| Chr02:11030000-19249999 | BG_GLEAN_10022225 | \| Symbols: ORF158 \| DNA/RNA polymerases superfamily protein \| chrM:235916-236392 FORWARD LENGTH=158 |
| Chr02:11030000-19249999 | BG_GLEAN_10022226 | _ |
| Chr02:11030000-19249999 | BG_GLEAN_10022227 | \| Symbols: \| unknown protein; FUNCTIONS IN: molecular_function unknown; INVOLVED IN: biological_process unknown; LOCATED IN: cellular_component unknown; EXPRESSED IN: leaf; Has 72 Blast hits to 72 proteins in 9 species: Archae - 0; Bacteria - 0; Metazoa - 0; Fungi - 0; Plants - 72; Viruses - 0; Other Eukaryotes - 0 (source: NCBI BLink). \| chr1:25011008-25012987 REVERSE LENGTH=659 |
| Chr02:11030000-19249999 | BG_GLEAN_10022228 | _ |
| Chr02:11030000-19249999 | BG_GLEAN_10022229 | \| Symbols: \| RING/U-box superfamily protein \| chr1:18205946-18206701 FORWARD LENGTH=251 |
| Chr02:11030000-19249999 | BG_GLEAN_10022230 | \| Symbols: ATRMA3, RMA3 \| RING membrane-anchor 3 \| chr4:13735576-13736307 FORWARD LENGTH=243 |
| Chr02:11030000-19249999 | BG_GLEAN_10022231 | \| Symbols: \| Plasma-membrane choline transporter family protein \| chr3:5193319-5196435 FORWARD LENGTH=700 |
| Chr02:11030000-19249999 | BG_GLEAN_10022232 | _ |
| Chr02:11030000-19249999 | BG_GLEAN_10022233 | \| Symbols: MLO11, ATMLO11 \| Seven transmembrane MLO family protein \| chr5:21823055-21826289 FORWARD LENGTH=573 |
| Chr02:11030000-19249999 | BG_GLEAN_10022234 | \| Symbols: MLO11, ATMLO11 \| Seven transmembrane MLO family protein \| chr5:21823055-21826289 FORWARD LENGTH=573 |
| Chr02:11030000-19249999 | BG_GLEAN_10022235 | _ |
| Chr02:11030000-19249999 | BG_GLEAN_10022236 | \| Symbols: \| Regulator of chromosome condensation (RCC1) family protein \| chr3:5209408-5211562 FORWARD LENGTH=488 |
| Chr02:11030000-19249999 | BG_GLEAN_10022237 | \| Symbols: \| CBS domain-containing protein \| chr5:21817416-21818723 FORWARD LENGTH=408 |
| Chr02:11030000-19249999 | BG_GLEAN_10022238 | \| Symbols: \| Aluminium induced protein with YGL and LRDR motifs \| chr4:13727665-13728683 REVERSE LENGTH=250 |
| Chr02:11030000-19249999 | BG_GLEAN_10022239 | \| Symbols: CRK8 \| cysteine-rich RLK (RECEPTOR-like protein kinase) 8 \| chr4:12129485-12134086 FORWARD LENGTH=1262 |
| Chr02:11030000-19249999 | BG_GLEAN_10022240 | \| Symbols: GCR2, GPCR \| G protein coupled receptor \| chr1:19709360-19711048 REVERSE LENGTH=410 |
| Chr02:11030000-19249999 | BG_GLEAN_10022241 | \| Symbols: PORB \| protochlorophyllide oxidoreductase B \| chr4:13725648-13727107 FORWARD LENGTH=401 |
| Chr02:11030000-19249999 | BG_GLEAN_10022242 | \| Symbols: \| Transducin/WD40 repeat-like superfamily protein \| chr3:5216630-5219868 REVERSE LENGTH=883 |
| Chr02:11030000-19249999 | BG_GLEAN_10022243 | \| Symbols: \| Protein of unknown function (DUF1218) \| chr4:13723903-13724872 FORWARD LENGTH=173 |
| Chr02:11030000-19249999 | BG_GLEAN_10022244 | \| Symbols: CIP7 \| COP1-interacting protein 7 \| chr4:13718817-13722736 FORWARD LENGTH=1058 |
| Chr02:11030000-19249999 | BG_GLEAN_10022246 | _ |
| Chr02:11030000-19249999 | BG_GLEAN_10022247 | \| Symbols: BGLU45 \| beta-glucosidase 45 \| chr1:22830035-22832813 FORWARD LENGTH=520 |
| Chr02:11030000-19249999 | BG_GLEAN_10022249 | \| Symbols: \| CONTAINS InterPro DOMAIN/s: Retrotransposon gag protein (InterPro:IPR005162); Has 707 Blast hits to 705 proteins in 25 species: Archae - 0; Bacteria - 0; Metazoa - 4; Fungi - 0; Plants - 703; Viruses - 0; Other Eukaryotes - 0 (source: NCBI BLink). \| chr1:7447690-7448403 REVERSE LENGTH=237 |
| Chr02:11030000-19249999 | BG_GLEAN_10022250 | _ |
| Chr02:11030000-19249999 | BG_GLEAN_10022251 | _ |
| Chr02:11030000-19249999 | BG_GLEAN_10022253 | \| Symbols: RD26, ANAC072 \| NAC (No Apical Meristem) domain transcriptional regulator superfamily protein \| chr4:13707928-13709013 REVERSE LENGTH=297 |
| Chr02:11030000-19249999 | BG_GLEAN_10022254 | _ |
| Chr02:11030000-19249999 | BG_GLEAN_10022255 | \| Symbols: \| unknown protein; Has 90 Blast hits to 90 proteins in 7 species: Archae - 0; Bacteria - 0; Metazoa - 0; Fungi - 0; Plants - 90; Viruses - 0; Other Eukaryotes - 0 (source: NCBI BLink). \| chr3:11612357-11612686 REVERSE LENGTH=109 |
| Chr02:11030000-19249999 | BG_GLEAN_10022256 | \| Symbols: ATNAC2, ANAC056, NARS1, NAC2 \| NAC domain containing protein 2 \| chr3:5243696-5245037 FORWARD LENGTH=364 |
| Chr02:11030000-19249999 | BG_GLEAN_10022257 | \| Symbols: \| unknown protein; FUNCTIONS IN: molecular_function unknown; INVOLVED IN: biological_process unknown; LOCATED IN: cellular_component unknown; Has 30201 Blast hits to 17322 proteins in 780 species: Archae - 12; Bacteria - 1396; Metazoa - 17338; Fungi - 3422; Plants - 5037; Viruses - 0; Other Eukaryotes - 2996 (source: NCBI BLink). \| chr3:5249221-5249541 FORWARD LENGTH=106 |
| Chr02:11030000-19249999 | BG_GLEAN_10022258 | \| Symbols: LCR78, PDF1.4 \| Arabidopsis defensin-like protein \| chr1:6781674-6782024 REVERSE LENGTH=78 |
| Chr02:11030000-19249999 | BG_GLEAN_10022259 | _ |
| Chr02:11030000-19249999 | BG_GLEAN_10022260 | _ |
| Chr02:11030000-19249999 | BG_GLEAN_10022261 | \| Symbols: CLUB, AtTRS130 \| CLUB \| chr5:22100056-22107695 FORWARD LENGTH=1259 |
| Chr02:11030000-19249999 | BG_GLEAN_10022262 | \| Symbols: \| CONTAINS InterPro DOMAIN/s: Retrotransposon gag protein (InterPro:IPR005162); BEST Arabidopsis thaliana protein match is: unknown protein (TAIR:AT5G48050.1); Has 648 Blast hits to 647 proteins in 29 species: Archae - 0; Bacteria - 0; Metazoa - 16; Fungi - 25; Plants - 607; Viruses - 0; Other Eukaryotes - 0 (source: NCBI BLink). \| chr1:12402283-12403209 FORWARD LENGTH=308 |
| Chr02:11030000-19249999 | BG_GLEAN_10022263 | \| Symbols: \| S-adenosyl-L-methionine-dependent methyltransferases superfamily protein \| chr5:22090680-22091998 FORWARD LENGTH=292 |
| Chr02:11030000-19249999 | BG_GLEAN_10022264 | \| Symbols: AHL, ATAHL, HL \| HAL2-like \| chr5:22086133-22087586 FORWARD LENGTH=373 |
| Chr02:11030000-19249999 | BG_GLEAN_10022265 | \| Symbols: \| Small nuclear ribonucleoprotein family protein \| chr4:1264726-1266253 FORWARD LENGTH=116 |
| Chr02:11030000-19249999 | BG_GLEAN_10022266 | \| Symbols: AHL, ATAHL, HL \| HAL2-like \| chr5:22086133-22087586 FORWARD LENGTH=373 |
| Chr02:11030000-19249999 | BG_GLEAN_10022267 | \| Symbols: emb1507 \| U5 small nuclear ribonucleoprotein helicase, putative \| chr1:7302591-7309914 REVERSE LENGTH=2171 |
| Chr02:11030000-19249999 | BG_GLEAN_10022268 | \| Symbols: \| U5 small nuclear ribonucleoprotein helicase \| chr2:17604330-17610848 FORWARD LENGTH=2172 |
| Chr02:11030000-19249999 | BG_GLEAN_10022269 | \| Symbols: THE1 \| protein kinase family protein \| chr5:22077313-22079880 REVERSE LENGTH=855 |
| Chr02:11030000-19249999 | BG_GLEAN_10022270 | \| Symbols: \| Late embryogenesis abundant (LEA) protein-related \| chr1:20463107-20464407 FORWARD LENGTH=347 |
| Chr02:11030000-19249999 | BG_GLEAN_10022271 | \| Symbols: THE1 \| protein kinase family protein \| chr5:22077313-22079880 REVERSE LENGTH=855 |
| Chr02:11030000-19249999 | BG_GLEAN_10022272 | \| Symbols: \| Late embryogenesis abundant (LEA) protein-related \| chr5:22075334-22076567 FORWARD LENGTH=337 |
| Chr02:11030000-19249999 | BG_GLEAN_10022273 | \| Symbols: LDL2 \| LSD1-like2 \| chr3:4479193-4481509 REVERSE LENGTH=746 |
| Chr02:11030000-19249999 | BG_GLEAN_10022274 | _ |
| Chr02:11030000-19249999 | BG_GLEAN_10022275 | \| Symbols: ULT1, ULT \| Developmental regulator, ULTRAPETALA \| chr4:13985753-13987050 FORWARD LENGTH=237 |
| Chr02:11030000-19249999 | BG_GLEAN_10022276 | _ |
| Chr02:11030000-19249999 | BG_GLEAN_10022277 | \| Symbols: \| Protein of unknown function (DUF300) \| chr4:11471126-11472269 REVERSE LENGTH=294 |
| Chr02:11030000-19249999 | BG_GLEAN_10022278 | \| Symbols: \| unknown protein; FUNCTIONS IN: molecular_function unknown; INVOLVED IN: biological_process unknown; LOCATED IN: cellular_component unknown; Has 24 Blast hits to 24 proteins in 11 species: Archae - 0; Bacteria - 0; Metazoa - 0; Fungi - 0; Plants - 24; Viruses - 0; Other Eukaryotes - 0 (source: NCBI BLink). \| chr3:4476296-4476611 FORWARD LENGTH=76 |
| Chr02:11030000-19249999 | BG_GLEAN_10022279 | \| Symbols: \| unknown protein; FUNCTIONS IN: molecular_function unknown; INVOLVED IN: biological_process unknown; LOCATED IN: cellular_component unknown; Has 24 Blast hits to 24 proteins in 11 species: Archae - 0; Bacteria - 0; Metazoa - 0; Fungi - 0; Plants - 24; Viruses - 0; Other Eukaryotes - 0 (source: NCBI BLink). \| chr3:4476296-4476611 FORWARD LENGTH=76 |
| Chr02:11030000-19249999 | BG_GLEAN_10022280 | \| Symbols: NEV, AGD5 \| ARF-GAP domain 5 \| chr5:22057262-22061066 REVERSE LENGTH=483 |
| Chr02:11030000-19249999 | BG_GLEAN_10022281 | \| Symbols: \| Protein of unknown function (DUF761) \| chr5:22054270-22055342 REVERSE LENGTH=326 |
| Chr02:11030000-19249999 | BG_GLEAN_10022282 | \| Symbols: ATM2, ATMYOS1, ATM4 \| myosin 2 \| chr5:22039606-22045592 REVERSE LENGTH=1220 |
| Chr02:11030000-19249999 | BG_GLEAN_10022283 | _ |
| Chr02:11030000-19249999 | BG_GLEAN_10022284 | \| Symbols: MRE11, ATMRE11 \| DNA repair and meiosis protein (Mre11) \| chr5:22032702-22037749 FORWARD LENGTH=720 |
| Chr02:11030000-19249999 | BG_GLEAN_10022285 | _ |
| Chr02:11030000-19249999 | BG_GLEAN_10022286 | _ |
| Chr02:11030000-19249999 | BG_GLEAN_10022287 | \| Symbols: \| Dynein light chain type 1 family protein \| chr4:13694032-13694517 FORWARD LENGTH=103 |
| Chr02:11030000-19249999 | BG_GLEAN_10022288 | \| Symbols: \| Protein of unknown function (DUF1223) \| chr5:22023339-22024461 REVERSE LENGTH=282 |
| Chr02:11030000-19249999 | BG_GLEAN_10022289 | \| Symbols: ORF158 \| DNA/RNA polymerases superfamily protein \| chrM:235916-236392 FORWARD LENGTH=158 |
| Chr02:11030000-19249999 | BG_GLEAN_10022290 | \| Symbols: ATMAMI, MAMI \| membrane-associated mannitol-induced \| chr5:21958356-21960367 FORWARD LENGTH=266 |
| Chr02:11030000-19249999 | BG_GLEAN_10022291 | \| Symbols: UBP14, TTN6, ATUBP14, PER1 \| ubiquitin-specific protease 14 \| chr3:7203001-7208340 REVERSE LENGTH=797 |
| Chr02:11030000-19249999 | BG_GLEAN_10022292 | _ |
| Chr02:11030000-19249999 | BG_GLEAN_10022294 | _ |
| Chr02:11030000-19249999 | BG_GLEAN_10022296 | \| Symbols: \| Met-10+ like family protein \| chr4:13687366-13690370 REVERSE LENGTH=619 |
| Chr02:11030000-19249999 | BG_GLEAN_10022297 | _ |
| Chr02:11030000-19249999 | BG_GLEAN_10022299 | \| Symbols: ATPHOS34, PHOS34 \| Adenine nucleotide alpha hydrolases-like superfamily protein \| chr4:13678860-13680717 REVERSE LENGTH=260 |
| Chr02:11030000-19249999 | BG_GLEAN_10022300 | _ |
| Chr02:11030000-19249999 | BG_GLEAN_10022301 | \| Symbols: \| B-box type zinc finger family protein \| chr4:13675853-13676616 FORWARD LENGTH=223 |
| Chr02:11030000-19249999 | BG_GLEAN_10022303 | _ |
| Chr02:11030000-19249999 | BG_GLEAN_10022304 | _ |
| Chr02:11030000-19249999 | BG_GLEAN_10022305 | _ |
| Chr02:11030000-19249999 | BG_GLEAN_10022306 | \| Symbols: \| Plant self-incompatibility protein S1 family \| chr4:9173165-9173650 REVERSE LENGTH=161 |
| Chr02:11030000-19249999 | BG_GLEAN_10022307 | \| Symbols: SPH1 \| S-protein homologue 1 \| chr4:9215680-9216135 REVERSE LENGTH=151 |
| Chr02:11030000-19249999 | BG_GLEAN_10022309 | _ |
| Chr02:11030000-19249999 | BG_GLEAN_10022310 | \| Symbols: \| unknown protein; Has 229 Blast hits to 229 proteins in 10 species: Archae - 0; Bacteria - 0; Metazoa - 1; Fungi - 0; Plants - 228; Viruses - 0; Other Eukaryotes - 0 (source: NCBI BLink). \| chr1:18018148-18018441 FORWARD LENGTH=97 |
| Chr02:11030000-19249999 | BG_GLEAN_10022311 | \| Symbols: CRK8 \| cysteine-rich RLK (RECEPTOR-like protein kinase) 8 \| chr4:12129485-12134086 FORWARD LENGTH=1262 |
| Chr02:11030000-19249999 | BG_GLEAN_10022312 | \| Symbols: ORF158 \| DNA/RNA polymerases superfamily protein \| chrM:235916-236392 FORWARD LENGTH=158 |
| Chr02:11030000-19249999 | BG_GLEAN_10022313 | _ |
| Chr02:11030000-19249999 | BG_GLEAN_10022314 | \| Symbols: \| MuDR family transposase \| chr1:23847756-23849915 FORWARD LENGTH=719 |
| Chr02:11030000-19249999 | BG_GLEAN_10022315 | \| Symbols: GH3.6, DFL1 \| Auxin-responsive GH3 family protein \| chr5:22131321-22133564 REVERSE LENGTH=612 |
| Chr02:11030000-19249999 | BG_GLEAN_10022316 | \| Symbols: \| plastid developmental protein DAG, putative \| chr1:3847273-3848938 FORWARD LENGTH=232 |
| Chr02:11030000-19249999 | BG_GLEAN_10022317 | \| Symbols: \| Protein of unknown function, DUF538 \| chr5:22152781-22154201 FORWARD LENGTH=161 |
| Chr02:11030000-19249999 | BG_GLEAN_10022319 | \| Symbols: \| Uncharacterised conserved protein (UCP012943) \| chr5:22156604-22157857 FORWARD LENGTH=297 |
| Chr02:11030000-19249999 | BG_GLEAN_10022320 | \| Symbols: \| mRNA capping enzyme family protein \| chr3:7221168-7223939 REVERSE LENGTH=370 |
| Chr02:11030000-19249999 | BG_GLEAN_10022321 | \| Symbols: CRK8 \| cysteine-rich RLK (RECEPTOR-like protein kinase) 8 \| chr4:12129485-12134086 FORWARD LENGTH=1262 |
| Chr02:11030000-19249999 | BG_GLEAN_10022323 | \| Symbols: \| MuDR family transposase \| chr1:23847756-23849915 FORWARD LENGTH=719 |
| Chr02:11030000-19249999 | BG_GLEAN_10022324 | \| Symbols: BGLU41 \| beta glucosidase 41 \| chr5:22167636-22170235 REVERSE LENGTH=535 |
| Chr02:11030000-19249999 | BG_GLEAN_10022325 | \| Symbols: ORF158 \| DNA/RNA polymerases superfamily protein \| chrM:235916-236392 FORWARD LENGTH=158 |
| Chr02:11030000-19249999 | BG_GLEAN_10022326 | _ |
| Chr02:11030000-19249999 | BG_GLEAN_10022327 | \| Symbols: \| Ribosomal protein L2 family \| chr4:17097613-17098656 FORWARD LENGTH=258 |
| Chr02:11030000-19249999 | BG_GLEAN_10022328 | _ |
| Chr02:11030000-19249999 | BG_GLEAN_10022329 | \| Symbols: \| RNA-binding (RRM/RBD/RNP motifs) family protein \| chr5:22171332-22172656 FORWARD LENGTH=156 |
| Chr02:11030000-19249999 | BG_GLEAN_10022330 | \| Symbols: AtOCT4, 4-Oct \| organic cation/carnitine transporter4 \| chr3:7225271-7228510 REVERSE LENGTH=526 |
| Chr02:11030000-19249999 | BG_GLEAN_10022331 | \| Symbols: AtOCT4, 4-Oct \| organic cation/carnitine transporter4 \| chr3:7225271-7228510 REVERSE LENGTH=526 |
| Chr02:11030000-19249999 | BG_GLEAN_10022332 | \| Symbols: PAT1 \| GRAS family transcription factor \| chr5:19522497-19524053 REVERSE LENGTH=490 |
| Chr02:11030000-19249999 | BG_GLEAN_10022334 | \| Symbols: CRLK1 \| Protein kinase superfamily protein \| chr5:22180480-22182698 FORWARD LENGTH=440 |
| Chr02:11030000-19249999 | BG_GLEAN_10022335 | \| Symbols: \| zinc finger protein-related \| chr5:22192607-22194260 REVERSE LENGTH=472 |
| Chr02:11030000-19249999 | BG_GLEAN_10022336 | \| Symbols: HTA10 \| histone H2A 10 \| chr1:18926948-18927443 FORWARD LENGTH=132 |
| Chr02:11030000-19249999 | BG_GLEAN_10022337 | \| Symbols: Fh5, ATFH5 \| formin homology5 \| chr5:22197856-22201649 REVERSE LENGTH=900 |
| Chr02:11030000-19249999 | BG_GLEAN_10022338 | \| Symbols: ATK3, KATC \| kinesin 3 \| chr5:22209912-22213843 FORWARD LENGTH=754 |
| Chr02:11030000-19249999 | BG_GLEAN_10022339 | \| Symbols: PQL1, PQL2 \| PsbQ-like 1 \| chr3:168478-169407 FORWARD LENGTH=220 |
| Chr02:11030000-19249999 | BG_GLEAN_10022340 | _ |
| Chr02:11030000-19249999 | BG_GLEAN_10022341 | _ |
| Chr02:11030000-19249999 | BG_GLEAN_10022342 | _ |
| Chr02:11030000-19249999 | BG_GLEAN_10022343 | \| Symbols: GPT2, ATGPT2 \| glucose-6-phosphate/phosphate translocator 2 \| chr1:22824527-22826459 FORWARD LENGTH=388 |
| Chr02:11030000-19249999 | BG_GLEAN_10022344 | \| Symbols: TSB2 \| tryptophan synthase beta-subunit 2 \| chr4:13586564-13588619 FORWARD LENGTH=475 |
| Chr02:11030000-19249999 | BG_GLEAN_10022345 | _ |
| Chr02:11030000-19249999 | BG_GLEAN_10022346 | \| Symbols: ORF158 \| DNA/RNA polymerases superfamily protein \| chrM:235916-236392 FORWARD LENGTH=158 |
| Chr02:11030000-19249999 | BG_GLEAN_10022350 | _ |
| Chr02:11030000-19249999 | BG_GLEAN_10022351 | _ |
| Chr02:11030000-19249999 | BG_GLEAN_10022353 | _ |
| Chr02:11030000-19249999 | BG_GLEAN_10022354 | _ |
| Chr02:11030000-19249999 | BG_GLEAN_10022356 | \| Symbols: DDL \| SMAD/FHA domain-containing protein \| chr3:7174695-7177600 REVERSE LENGTH=314 |
| Chr02:11030000-19249999 | BG_GLEAN_10022357 | _ |
| Chr02:11030000-19249999 | BG_GLEAN_10022358 | _ |
| Chr02:11030000-19249999 | BG_GLEAN_10022359 | \| Symbols: ATSGP1, SGP1 \| Ras-related small GTP-binding family protein \| chr5:22276611-22278328 REVERSE LENGTH=288 |
| Chr02:11030000-19249999 | BG_GLEAN_10022360 | \| Symbols: CHL \| chloroplastic lipocalin \| chr3:17656778-17658269 REVERSE LENGTH=353 |
| Chr02:11030000-19249999 | BG_GLEAN_10022361 | \| Symbols: \| unknown protein; Has 1807 Blast hits to 1807 proteins in 277 species: Archae - 0; Bacteria - 0; Metazoa - 736; Fungi - 347; Plants - 385; Viruses - 0; Other Eukaryotes - 339 (source: NCBI BLink). \| chr5:22280755-22281363 REVERSE LENGTH=202 |
| Chr02:11030000-19249999 | BG_GLEAN_10022362 | \| Symbols: TOR1, SPR2, CN \| ARM repeat superfamily protein \| chr4:13581581-13585070 REVERSE LENGTH=864 |
| Chr02:11030000-19249999 | BG_GLEAN_10022363 | \| Symbols: TOR1, SPR2, CN \| ARM repeat superfamily protein \| chr4:13581581-13585070 REVERSE LENGTH=864 |
| Chr02:11030000-19249999 | BG_GLEAN_10022364 | \| Symbols: \| FUNCTIONS IN: molecular_function unknown; INVOLVED IN: biological_process unknown; LOCATED IN: membrane; EXPRESSED IN: 14 plant structures; EXPRESSED DURING: 4 anthesis, C globular stage, F mature embryo stage, petal differentiation and expansion stage, E expanded cotyledon stage; CONTAINS InterPro DOMAIN/s: Aminotransferase-like, plant mobile domain (InterPro:IPR019557), Protein of unknown function DUF716 (InterPro:IPR006904); BEST Arabidopsis thaliana protein match is: Aminotransferase-like, plant mobile domain family protein (TAIR:AT1G51538.1); Has 16736 Blast hits to 9656 proteins in 576 species: Archae - 4; Bacteria - 1182; Metazoa - 7098; Fungi - 2631; Plants - 1178; Viruses - 174; Other Eukaryotes - 4469 (source: NCBI BLink). \| chr1:11552926-11558608 FORWARD LENGTH=1206 |
| Chr02:11030000-19249999 | BG_GLEAN_10022365 | _ |
| Chr02:11030000-19249999 | BG_GLEAN_10022366 | \| Symbols: ATCXXS1, CXXS1 \| C-terminal cysteine residue is changed to a serine 1 \| chr1:3874518-3875311 FORWARD LENGTH=118 |
| Chr02:11030000-19249999 | BG_GLEAN_10022368 | \| Symbols: ILR3, bHLH105 \| basic helix-loop-helix (bHLH) DNA-binding superfamily protein \| chr5:22217270-22218993 FORWARD LENGTH=234 |
| Chr02:11030000-19249999 | BG_GLEAN_10022369 | \| Symbols: ATATG18F, ATG18F, G18F \| homolog of yeast autophagy 18 (ATG18) F \| chr5:22233977-22236804 REVERSE LENGTH=763 |
| Chr02:11030000-19249999 | BG_GLEAN_10022370 | \| Symbols: ATATG18G, ATG18G \| homolog of yeast autophagy 18 (ATG18) G \| chr1:836155-840362 FORWARD LENGTH=959 |
| Chr02:11030000-19249999 | BG_GLEAN_10022371 | _ |
| Chr02:11030000-19249999 | BG_GLEAN_10022372 | _ |
| Chr02:11030000-19249999 | BG_GLEAN_10022373 | _ |
| Chr02:11030000-19249999 | BG_GLEAN_10022374 | \| Symbols: FIE, FIS3, FIE1 \| Transducin/WD40 repeat-like superfamily protein \| chr3:7249064-7252254 REVERSE LENGTH=369 |
| Chr02:11030000-19249999 | BG_GLEAN_10022375 | _ |
| Chr02:11030000-19249999 | BG_GLEAN_10022376 | _ |
| Chr02:11030000-19249999 | BG_GLEAN_10022377 | \| Symbols: \| Tetratricopeptide repeat (TPR)-like superfamily protein \| chr4:2435007-2439344 REVERSE LENGTH=821 |
| Chr02:11030000-19249999 | BG_GLEAN_10022379 | _ |
| Chr02:11030000-19249999 | BG_GLEAN_10022380 | _ |
| Chr02:11030000-19249999 | BG_GLEAN_10022381 | \| Symbols: \| ABC transporter family protein \| chr3:10593921-10598775 REVERSE LENGTH=1240 |
| Chr02:11030000-19249999 | BG_GLEAN_10022382 | \| Symbols: NDR1 \| Late embryogenesis abundant (LEA) hydroxyproline-rich glycoprotein family \| chr3:7194877-7195536 FORWARD LENGTH=219 |
| Chr02:11030000-19249999 | BG_GLEAN_10022383 | \| Symbols: \| Transport protein particle (TRAPP) component \| chr5:22242080-22243477 FORWARD LENGTH=186 |
| Chr02:11030000-19249999 | BG_GLEAN_10022384 | _ |
| Chr02:11030000-19249999 | BG_GLEAN_10022385 | \| Symbols: UBQ10 \| polyubiquitin 10 \| chr4:2718559-2719932 FORWARD LENGTH=457 |
| Chr02:11030000-19249999 | BG_GLEAN_10022386 | \| Symbols: \| CONTAINS InterPro DOMAIN/s: DDRGK domain (InterPro:IPR019153); Has 30201 Blast hits to 17322 proteins in 780 species: Archae - 12; Bacteria - 1396; Metazoa - 17338; Fungi - 3422; Plants - 5037; Viruses - 0; Other Eukaryotes - 2996 (source: NCBI BLink). \| chr4:13602210-13604227 REVERSE LENGTH=298 |
| Chr02:11030000-19249999 | BG_GLEAN_10022387 | _ |
| Chr02:11030000-19249999 | BG_GLEAN_10022388 | _ |
| Chr02:11030000-19249999 | BG_GLEAN_10022389 | \| Symbols: COBL10 \| COBRA-like protein 10 precursor \| chr3:7188063-7190416 REVERSE LENGTH=672 |
| Chr02:11030000-19249999 | BG_GLEAN_10022390 | \| Symbols: ENODL9, AtENODL9 \| early nodulin-like protein 9 \| chr3:7186754-7187453 REVERSE LENGTH=203 |
| Chr02:11030000-19249999 | BG_GLEAN_10022391 | \| Symbols: ATPDIL5-4, ATPDI7, PDI7, PDIL5-4 \| PDI-like 5-4 \| chr4:13589156-13593335 FORWARD LENGTH=480 |
| Chr02:11030000-19249999 | BG_GLEAN_10022393 | _ |
| Chr02:11030000-19249999 | BG_GLEAN_10022394 | \| Symbols: ETFALPHA \| electron transfer flavoprotein alpha \| chr1:18878038-18879939 REVERSE LENGTH=363 |
| Chr02:11030000-19249999 | BG_GLEAN_10022395 | _ |
| Chr02:11030000-19249999 | BG_GLEAN_10022396 | \| Symbols: \| unknown protein; BEST Arabidopsis thaliana protein match is: unknown protein (TAIR:AT1G50930.1); Has 53 Blast hits to 53 proteins in 10 species: Archae - 0; Bacteria - 0; Metazoa - 0; Fungi - 0; Plants - 53; Viruses - 0; Other Eukaryotes - 0 (source: NCBI BLink). \| chr5:22254195-22255489 REVERSE LENGTH=165 |
| Chr02:11030000-19249999 | BG_GLEAN_10022397 | \| Symbols: CRK8 \| cysteine-rich RLK (RECEPTOR-like protein kinase) 8 \| chr4:12129485-12134086 FORWARD LENGTH=1262 |
| Chr02:11030000-19249999 | BG_GLEAN_10022398 | \| Symbols: PDF2 \| protodermal factor 2 \| chr4:2476970-2480090 REVERSE LENGTH=743 |
| Chr02:11030000-19249999 | BG_GLEAN_10022399 | \| Symbols: \| unknown protein; Has 266 Blast hits to 264 proteins in 44 species: Archae - 0; Bacteria - 0; Metazoa - 58; Fungi - 0; Plants - 199; Viruses - 0; Other Eukaryotes - 9 (source: NCBI BLink). \| chr3:5104582-5108279 FORWARD LENGTH=562 |
| Chr02:11030000-19249999 | BG_GLEAN_10022400 | _ |
| Chr02:11030000-19249999 | BG_GLEAN_10022401 | _ |
| Chr02:11030000-19249999 | BG_GLEAN_10022402 | _ |
| Chr02:11030000-19249999 | BG_GLEAN_10022403 | _ |
| Chr02:11030000-19249999 | BG_GLEAN_10022404 | _ |
| Chr02:11030000-19249999 | BG_GLEAN_10022405 | _ |
| Chr02:11030000-19249999 | BG_GLEAN_10022406 | _ |
| Chr02:11030000-19249999 | BG_GLEAN_10022408 | \| Symbols: \| unknown protein; BEST Arabidopsis thaliana protein match is: unknown protein (TAIR:AT1G40129.1); Has 30201 Blast hits to 17322 proteins in 780 species: Archae - 12; Bacteria - 1396; Metazoa - 17338; Fungi - 3422; Plants - 5037; Viruses - 0; Other Eukaryotes - 2996 (source: NCBI BLink). \| chr4:4172609-4175227 REVERSE LENGTH=330 |
| Chr02:11030000-19249999 | BG_GLEAN_10022409 | \| Symbols: \| unknown protein; Has 754 Blast hits to 165 proteins in 64 species: Archae - 0; Bacteria - 48; Metazoa - 26; Fungi - 25; Plants - 36; Viruses - 0; Other Eukaryotes - 619 (source: NCBI BLink). \| chr3:7154178-7156382 REVERSE LENGTH=458 |
| Chr02:11030000-19249999 | BG_GLEAN_10022410 | _ |
| Chr02:11030000-19249999 | BG_GLEAN_10022411 | \| Symbols: ORF158 \| DNA/RNA polymerases superfamily protein \| chrM:235916-236392 FORWARD LENGTH=158 |
| Chr02:11030000-19249999 | BG_GLEAN_10022412 | \| Symbols: CRK8 \| cysteine-rich RLK (RECEPTOR-like protein kinase) 8 \| chr4:12129485-12134086 FORWARD LENGTH=1262 |
| Chr02:11030000-19249999 | BG_GLEAN_10022413 | _ |
| Chr02:11030000-19249999 | BG_GLEAN_10022415 | _ |
| Chr02:11030000-19249999 | BG_GLEAN_10022416 | \| Symbols: \| unknown protein; FUNCTIONS IN: molecular_function unknown; LOCATED IN: vacuole; BEST Arabidopsis thaliana protein match is: unknown protein (TAIR:AT5G54870.1); Has 1807 Blast hits to 1807 proteins in 277 species: Archae - 0; Bacteria - 0; Metazoa - 736; Fungi - 347; Plants - 385; Viruses - 0; Other Eukaryotes - 339 (source: NCBI BLink). \| chr4:13568604-13571381 REVERSE LENGTH=523 |
| Chr02:11030000-19249999 | BG_GLEAN_10022417 | _ |
| Chr02:11030000-19249999 | BG_GLEAN_10022418 | _ |
| Chr02:11030000-19249999 | BG_GLEAN_10022420 | \| Symbols: \| unknown protein; FUNCTIONS IN: molecular_function unknown; INVOLVED IN: biological_process unknown; LOCATED IN: vacuole; EXPRESSED IN: 24 plant structures; EXPRESSED DURING: 15 growth stages; BEST Arabidopsis thaliana protein match is: unknown protein (TAIR:AT4G27020.1); Has 1807 Blast hits to 1807 proteins in 277 species: Archae - 0; Bacteria - 0; Metazoa - 736; Fungi - 347; Plants - 385; Viruses - 0; Other Eukaryotes - 339 (source: NCBI BLink). \| chr5:22289149-22291604 FORWARD LENGTH=531 |
| Chr02:11030000-19249999 | BG_GLEAN_10022421 | _ |
| Chr02:11030000-19249999 | BG_GLEAN_10022422 | _ |
| Chr02:11030000-19249999 | BG_GLEAN_10022423 | _ |
| Chr02:11030000-19249999 | BG_GLEAN_10022424 | \| Symbols: CYP716A1 \| cytochrome P450, family 716, subfamily A, polypeptide 1 \| chr5:14195377-14197613 FORWARD LENGTH=477 |
| Chr02:11030000-19249999 | BG_GLEAN_10022425 | \| Symbols: \| Transmembrane proteins 14C \| chr3:7160884-7161991 FORWARD LENGTH=119 |
| Chr02:11030000-19249999 | BG_GLEAN_10022426 | \| Symbols: \| RNA-binding CRS1 / YhbY (CRM) domain-containing protein \| chr5:22293346-22294909 FORWARD LENGTH=358 |
| Chr02:11030000-19249999 | BG_GLEAN_10022427 | \| Symbols: \| RNA-binding CRS1 / YhbY (CRM) domain-containing protein \| chr5:22293346-22294909 FORWARD LENGTH=358 |
| Chr02:11030000-19249999 | BG_GLEAN_10022428 | \| Symbols: ATPAP18, PAP18 \| purple acid phosphatase 18 \| chr3:7157926-7160244 FORWARD LENGTH=437 |
| Chr02:11030000-19249999 | BG_GLEAN_10022429 | \| Symbols: ORF158 \| DNA/RNA polymerases superfamily protein \| chrM:235916-236392 FORWARD LENGTH=158 |
| Chr02:11030000-19249999 | BG_GLEAN_10022430 | _ |
| Chr02:11030000-19249999 | BG_GLEAN_10022431 | \| Symbols: \| DTW domain-containing protein \| chr5:22291920-22293104 FORWARD LENGTH=394 |
| Chr02:11030000-19249999 | BG_GLEAN_10022432 | _ |
| Chr02:11030000-19249999 | BG_GLEAN_10022433 | \| Symbols: \| heat shock protein 70 (Hsp 70) family protein \| chr1:3921056-3924347 FORWARD LENGTH=763 |
| Chr02:11030000-19249999 | BG_GLEAN_10022434 | \| Symbols: \| Polyketide cyclase/dehydrase and lipid transport superfamily protein \| chr1:5152465-5153035 REVERSE LENGTH=155 |
| Chr02:11030000-19249999 | BG_GLEAN_10022435 | \| Symbols: ORF145A \| Gag-Pol-related retrotransposon family protein \| chrM:89617-90054 REVERSE LENGTH=145 |
| Chr02:11030000-19249999 | BG_GLEAN_10022436 | \| Symbols: CRK8 \| cysteine-rich RLK (RECEPTOR-like protein kinase) 8 \| chr4:12129485-12134086 FORWARD LENGTH=1262 |
| Chr02:11030000-19249999 | BG_GLEAN_10022437 | _ |
| Chr02:11030000-19249999 | BG_GLEAN_10022438 | _ |
| Chr02:11030000-19249999 | BG_GLEAN_10022439 | \| Symbols: \| FUNCTIONS IN: molecular_function unknown; INVOLVED IN: biological_process unknown; LOCATED IN: cellular_component unknown; CONTAINS InterPro DOMAIN/s: mRNA splicing factor, Cwf21 (InterPro:IPR013170); Has 30201 Blast hits to 17322 proteins in 780 species: Archae - 12; Bacteria - 1396; Metazoa - 17338; Fungi - 3422; Plants - 5037; Viruses - 0; Other Eukaryotes - 2996 (source: NCBI BLink). \| chr3:18387504-18389279 REVERSE LENGTH=591 |
| Chr02:11030000-19249999 | BG_GLEAN_10022440 | \| Symbols: \| Polyketide cyclase/dehydrase and lipid transport superfamily protein \| chr1:5152465-5153035 REVERSE LENGTH=155 |
| Chr02:11030000-19249999 | BG_GLEAN_10022441 | _ |
| Chr02:11030000-19249999 | BG_GLEAN_10022442 | _ |
| Chr02:11030000-19249999 | BG_GLEAN_10022443 | _ |
| Chr02:11030000-19249999 | BG_GLEAN_10022445 | _ |
| Chr02:11030000-19249999 | BG_GLEAN_10022446 | _ |
| Chr02:11030000-19249999 | BG_GLEAN_10022447 | _ |
| Chr02:11030000-19249999 | BG_GLEAN_10022448 | _ |
| Chr02:11030000-19249999 | BG_GLEAN_10022449 | \| Symbols: ORF158 \| DNA/RNA polymerases superfamily protein \| chrM:235916-236392 FORWARD LENGTH=158 |
| Chr02:11030000-19249999 | BG_GLEAN_10022450 | _ |
| Chr02:11030000-19249999 | BG_GLEAN_10022451 | \| Symbols: \| Leucine-rich repeat protein kinase family protein \| chr1:3612228-3614343 FORWARD LENGTH=663 |
| Chr02:11030000-19249999 | BG_GLEAN_10022453 | \| Symbols: CRK8 \| cysteine-rich RLK (RECEPTOR-like protein kinase) 8 \| chr4:12129485-12134086 FORWARD LENGTH=1262 |
| Chr02:11030000-19249999 | BG_GLEAN_10022454 | _ |
| Chr02:11030000-19249999 | BG_GLEAN_10022455 | _ |
| Chr02:11030000-19249999 | BG_GLEAN_10022456 | \| Symbols: SIK1 \| Protein kinase superfamily protein \| chr1:26020298-26026119 REVERSE LENGTH=836 |
| Chr02:11030000-19249999 | BG_GLEAN_10022457 | \| Symbols: CRK8 \| cysteine-rich RLK (RECEPTOR-like protein kinase) 8 \| chr4:12129485-12134086 FORWARD LENGTH=1262 |
| Chr02:11030000-19249999 | BG_GLEAN_10022458 | \| Symbols: CRK8 \| cysteine-rich RLK (RECEPTOR-like protein kinase) 8 \| chr4:12129485-12134086 FORWARD LENGTH=1262 |
| Chr02:11030000-19249999 | BG_GLEAN_10022459 | \| Symbols: \| CONTAINS InterPro DOMAIN/s: Retrotransposon gag protein (InterPro:IPR005162); Has 707 Blast hits to 705 proteins in 25 species: Archae - 0; Bacteria - 0; Metazoa - 4; Fungi - 0; Plants - 703; Viruses - 0; Other Eukaryotes - 0 (source: NCBI BLink). \| chr1:7447690-7448403 REVERSE LENGTH=237 |
| Chr02:11030000-19249999 | BG_GLEAN_10022460 | _ |
| Chr02:11030000-19249999 | BG_GLEAN_10022461 | _ |
| Chr02:11030000-19249999 | BG_GLEAN_10022462 | _ |
| Chr02:11030000-19249999 | BG_GLEAN_10022463 | _ |
| Chr02:11030000-19249999 | BG_GLEAN_10022464 | _ |
| Chr02:11030000-19249999 | BG_GLEAN_10022465 | _ |
| Chr02:11030000-19249999 | BG_GLEAN_10022467 | \| Symbols: \| Polyketide cyclase/dehydrase and lipid transport superfamily protein \| chr1:5152465-5153035 REVERSE LENGTH=155 |
| Chr02:11030000-19249999 | BG_GLEAN_10022468 | _ |
| Chr02:11030000-19249999 | BG_GLEAN_10022471 | _ |
| Chr02:11030000-19249999 | BG_GLEAN_10022472 | _ |
| Chr02:11030000-19249999 | BG_GLEAN_10022473 | _ |
| Chr02:11030000-19249999 | BG_GLEAN_10022474 | _ |
| Chr02:11030000-19249999 | BG_GLEAN_10022475 | \| Symbols: \| unknown protein; BEST Arabidopsis thaliana protein match is: unknown protein (TAIR:AT1G40129.1); Has 30201 Blast hits to 17322 proteins in 780 species: Archae - 12; Bacteria - 1396; Metazoa - 17338; Fungi - 3422; Plants - 5037; Viruses - 0; Other Eukaryotes - 2996 (source: NCBI BLink). \| chr4:4172609-4175227 REVERSE LENGTH=330 |
| Chr02:11030000-19249999 | BG_GLEAN_10022476 | _ |
| Chr02:11030000-19249999 | BG_GLEAN_10022478 | \| Symbols: \| zinc knuckle (CCHC-type) family protein \| chr4:422732-424580 REVERSE LENGTH=488 |
| Chr02:11030000-19249999 | BG_GLEAN_10022479 | _ |
| Chr02:11030000-19249999 | BG_GLEAN_10022480 | _ |
| Chr02:11030000-19249999 | BG_GLEAN_10022481 | \| Symbols: \| Polyketide cyclase/dehydrase and lipid transport superfamily protein \| chr1:5152465-5153035 REVERSE LENGTH=155 |
| Chr02:11030000-19249999 | BG_GLEAN_10022483 | \| Symbols: \| RNase H family protein \| chr1:8520834-8522737 FORWARD LENGTH=353 |
| Chr02:11030000-19249999 | BG_GLEAN_10022484 | _ |
| Chr02:11030000-19249999 | BG_GLEAN_10022485 | _ |
| Chr02:11030000-19249999 | BG_GLEAN_10022486 | \| Symbols: \| CONTAINS InterPro DOMAIN/s: Retrotransposon gag protein (InterPro:IPR005162); Has 707 Blast hits to 705 proteins in 25 species: Archae - 0; Bacteria - 0; Metazoa - 4; Fungi - 0; Plants - 703; Viruses - 0; Other Eukaryotes - 0 (source: NCBI BLink). \| chr1:7447690-7448403 REVERSE LENGTH=237 |
| Chr02:11030000-19249999 | BG_GLEAN_10022487 | _ |
| Chr02:11030000-19249999 | BG_GLEAN_10022491 | \| Symbols: \| Polyketide cyclase/dehydrase and lipid transport superfamily protein \| chr1:5154775-5155512 REVERSE LENGTH=139 |
| Chr02:11030000-19249999 | BG_GLEAN_10022492 | _ |
| Chr02:11030000-19249999 | BG_GLEAN_10022493 | _ |
| Chr02:11030000-19249999 | BG_GLEAN_10022497 | \| Symbols: ATSBT1.1, SBTI1.1 \| subtilase family protein \| chr1:310332-313011 FORWARD LENGTH=774 |
| Chr02:11030000-19249999 | BG_GLEAN_10022500 | _ |
| Chr02:11030000-19249999 | BG_GLEAN_10022502 | \| Symbols: ATSBT1.1, SBTI1.1 \| subtilase family protein \| chr1:310332-313011 FORWARD LENGTH=774 |
| Chr02:11030000-19249999 | BG_GLEAN_10022504 | _ |
| Chr02:11030000-19249999 | BG_GLEAN_10022505 | _ |
| Chr02:11030000-19249999 | BG_GLEAN_10022506 | _ |
| Chr02:11030000-19249999 | BG_GLEAN_10022507 | _ |
| Chr02:11030000-19249999 | BG_GLEAN_10022508 | _ |
| Chr02:11030000-19249999 | BG_GLEAN_10022509 | _ |
| Chr02:11030000-19249999 | BG_GLEAN_10022510 | _ |
| Chr02:11030000-19249999 | BG_GLEAN_10022511 | \| Symbols: \| DEAD/DEAH box RNA helicase family protein \| chr2:193950-199056 REVERSE LENGTH=973 |
| Chr02:11030000-19249999 | BG_GLEAN_10022512 | _ |
| Chr02:11030000-19249999 | BG_GLEAN_10022513 | \| Symbols: BGAL7 \| beta-galactosidase 7 \| chr5:7010536-7013994 FORWARD LENGTH=826 |
| Chr02:11030000-19249999 | BG_GLEAN_10022514 | \| Symbols: BGAL7 \| beta-galactosidase 7 \| chr5:7010536-7013994 FORWARD LENGTH=826 |
| Chr02:11030000-19249999 | BG_GLEAN_10022515 | _ |
| Chr02:11030000-19249999 | BG_GLEAN_10022516 | _ |
| Chr02:11030000-19249999 | BG_GLEAN_10022517 | _ |
| Chr02:11030000-19249999 | BG_GLEAN_10022518 | \| Symbols: MLP31 \| MLP-like protein 31 \| chr1:26713170-26714014 REVERSE LENGTH=171 |
| Chr02:11030000-19249999 | BG_GLEAN_10022519 | \| Symbols: MLP31 \| MLP-like protein 31 \| chr1:26713170-26714014 REVERSE LENGTH=171 |
| Chr02:11030000-19249999 | BG_GLEAN_10022520 | \| Symbols: MLP28 \| MLP-like protein 28 \| chr1:26710203-26711395 REVERSE LENGTH=335 |
| Chr02:11030000-19249999 | BG_GLEAN_10022521 | _ |
| Chr02:11030000-19249999 | BG_GLEAN_10022522 | _ |
| Chr02:11030000-19249999 | BG_GLEAN_10022523 | _ |
| Chr02:11030000-19249999 | BG_GLEAN_10022525 | _ |
| Chr02:11030000-19249999 | BG_GLEAN_10022526 | _ |
| Chr02:11030000-19249999 | BG_GLEAN_10022527 | \| Symbols: \| Yos1-like protein \| chr2:15891900-15892136 REVERSE LENGTH=78 |
| Chr02:11030000-19249999 | BG_GLEAN_10022528 | _ |
| Chr02:11030000-19249999 | BG_GLEAN_10022529 | _ |
| Chr02:11030000-19249999 | BG_GLEAN_10022530 | \| Symbols: ORF158 \| DNA/RNA polymerases superfamily protein \| chrM:235916-236392 FORWARD LENGTH=158 |
| Chr02:11030000-19249999 | BG_GLEAN_10022531 | _ |
| Chr02:11030000-19249999 | BG_GLEAN_10022532 | \| Symbols: XSP1 \| xylem serine peptidase 1 \| chr4:93935-97289 FORWARD LENGTH=749 |
| Chr02:11030000-19249999 | BG_GLEAN_10022533 | \| Symbols: CRK8 \| cysteine-rich RLK (RECEPTOR-like protein kinase) 8 \| chr4:12129485-12134086 FORWARD LENGTH=1262 |
| Chr02:11030000-19249999 | BG_GLEAN_10022534 | \| Symbols: CRK8 \| cysteine-rich RLK (RECEPTOR-like protein kinase) 8 \| chr4:12129485-12134086 FORWARD LENGTH=1262 |
| Chr02:11030000-19249999 | BG_GLEAN_10022535 | \| Symbols: CRK8 \| cysteine-rich RLK (RECEPTOR-like protein kinase) 8 \| chr4:12129485-12134086 FORWARD LENGTH=1262 |
| Chr02:11030000-19249999 | BG_GLEAN_10022537 | _ |
| Chr02:11030000-19249999 | BG_GLEAN_10022538 | _ |
| Chr02:11030000-19249999 | BG_GLEAN_10022541 | \| Symbols: XSP1 \| xylem serine peptidase 1 \| chr4:93935-97289 FORWARD LENGTH=749 |
| Chr02:11030000-19249999 | BG_GLEAN_10022542 | _ |
| Chr02:11030000-19249999 | BG_GLEAN_10022543 | _ |
| Chr02:11030000-19249999 | BG_GLEAN_10022544 | _ |
| Chr02:11030000-19249999 | BG_GLEAN_10022545 | _ |
| Chr02:11030000-19249999 | BG_GLEAN_10022546 | _ |
| Chr02:11030000-19249999 | BG_GLEAN_10022547 | \| Symbols: \| zinc knuckle (CCHC-type) family protein \| chr4:422732-424580 REVERSE LENGTH=488 |
| Chr02:11030000-19249999 | BG_GLEAN_10022548 | _ |
| Chr02:11030000-19249999 | BG_GLEAN_10022549 | \| Symbols: scpl50 \| serine carboxypeptidase-like 50 \| chr1:5168613-5169947 FORWARD LENGTH=444 |
| Chr02:11030000-19249999 | BG_GLEAN_10022550 | \| Symbols: \| unknown protein; FUNCTIONS IN: molecular_function unknown; INVOLVED IN: biological_process unknown; LOCATED IN: endomembrane system; EXPRESSED IN: 22 plant structures; EXPRESSED DURING: 13 growth stages. \| chr1:5167354-5168151 REVERSE LENGTH=132 |
| Chr02:11030000-19249999 | BG_GLEAN_10022551 | \| Symbols: MPK9 \| MAP kinase 9 \| chr3:6174800-6178150 FORWARD LENGTH=510 |
| Chr02:11030000-19249999 | BG_GLEAN_10022552 | _ |
| Chr02:11030000-19249999 | BG_GLEAN_10022553 | _ |
| Chr02:11030000-19249999 | BG_GLEAN_10022555 | \| Symbols: ORF158 \| DNA/RNA polymerases superfamily protein \| chrM:235916-236392 FORWARD LENGTH=158 |
| Chr02:11030000-19249999 | BG_GLEAN_10022556 | \| Symbols: CRK8 \| cysteine-rich RLK (RECEPTOR-like protein kinase) 8 \| chr4:12129485-12134086 FORWARD LENGTH=1262 |
| Chr02:11030000-19249999 | BG_GLEAN_10022557 | \| Symbols: NUDX25 \| nudix hydrolase homolog 25 \| chr1:10582700-10583821 FORWARD LENGTH=175 |
| Chr02:11030000-19249999 | BG_GLEAN_10022558 | _ |
| Chr02:11030000-19249999 | BG_GLEAN_10022559 | _ |
| Chr02:11030000-19249999 | BG_GLEAN_10022560 | _ |
| Chr02:11030000-19249999 | BG_GLEAN_10022562 | _ |
| Chr02:11030000-19249999 | BG_GLEAN_10022564 | \| Symbols: ATHB17, ATHB-17, HB17 \| homeobox-leucine zipper protein 17 \| chr2:187798-190369 REVERSE LENGTH=275 |
| Chr02:11030000-19249999 | BG_GLEAN_10022565 | _ |
| Chr02:11030000-19249999 | BG_GLEAN_10022566 | _ |
| Chr02:11030000-19249999 | BG_GLEAN_10022567 | _ |
| Chr02:11030000-19249999 | BG_GLEAN_10022568 | \| Symbols: CLE16 \| CLAVATA3/ESR-RELATED 16 \| chr2:228926-229237 REVERSE LENGTH=103 |
| Chr02:11030000-19249999 | BG_GLEAN_10022569 | \| Symbols: CRK8 \| cysteine-rich RLK (RECEPTOR-like protein kinase) 8 \| chr4:12129485-12134086 FORWARD LENGTH=1262 |
| Chr02:11030000-19249999 | BG_GLEAN_10022570 | _ |
| Chr02:11030000-19249999 | BG_GLEAN_10022571 | _ |
| Chr02:11030000-19249999 | BG_GLEAN_10022572 | _ |
| Chr02:11030000-19249999 | BG_GLEAN_10022573 | _ |
| Chr02:11030000-19249999 | BG_GLEAN_10022574 | \| Symbols: \| Ribonuclease H-like superfamily protein \| chr4:14333528-14335255 FORWARD LENGTH=575 |
| Chr02:11030000-19249999 | BG_GLEAN_10022575 | \| Symbols: ACYB-2 \| Cytochrome b561/ferric reductase transmembrane protein family \| chr4:13053887-13055518 REVERSE LENGTH=239 |
| Chr02:11030000-19249999 | BG_GLEAN_10022577 | _ |
| Chr02:11030000-19249999 | BG_GLEAN_10022583 | _ |
| Chr02:11030000-19249999 | BG_GLEAN_10022584 | _ |
| Chr02:11030000-19249999 | BG_GLEAN_10022586 | _ |
| Chr02:11030000-19249999 | BG_GLEAN_10022587 | \| Symbols: XTR2, EXGT-A2, ATXTH28, XTH28 \| xyloglucan endotransglucosylase/hydrolase 28 \| chr1:5066806-5068466 REVERSE LENGTH=332 |
| Chr02:11030000-19249999 | BG_GLEAN_10022589 | _ |
| Chr02:11030000-19249999 | BG_GLEAN_10022590 | \| Symbols: ORF158 \| DNA/RNA polymerases superfamily protein \| chrM:235916-236392 FORWARD LENGTH=158 |
| Chr02:11030000-19249999 | BG_GLEAN_10022591 | _ |
| Chr02:11030000-19249999 | BG_GLEAN_10022592 | _ |
| Chr02:11030000-19249999 | BG_GLEAN_10022593 | _ |
| Chr02:11030000-19249999 | BG_GLEAN_10022594 | _ |
| Chr02:11030000-19249999 | BG_GLEAN_10022595 | \| Symbols: EMB260, EMB2421 \| FAD/NAD(P)-binding oxidoreductase family protein \| chr1:8635416-8638866 FORWARD LENGTH=709 |
| Chr02:11030000-19249999 | BG_GLEAN_10022597 | \| Symbols: ASB1, TRP4, WEI7 \| anthranilate synthase beta subunit 1 \| chr1:8837430-8839478 REVERSE LENGTH=276 |
| Chr02:11030000-19249999 | BG_GLEAN_10022598 | \| Symbols: \| hydroxyproline-rich glycoprotein family protein \| chr1:5062168-5064697 REVERSE LENGTH=601 |
| Chr02:11030000-19249999 | BG_GLEAN_10022599 | \| Symbols: \| hydroxyproline-rich glycoprotein family protein \| chr1:5062168-5064697 REVERSE LENGTH=601 |
| Chr02:11030000-19249999 | BG_GLEAN_10022600 | \| Symbols: \| DNAse I-like superfamily protein \| chr1:16528880-16531065 REVERSE LENGTH=626 |
| Chr02:11030000-19249999 | BG_GLEAN_10022601 | _ |
| Chr02:11030000-19249999 | BG_GLEAN_10022602 | _ |
| Chr02:11030000-19249999 | BG_GLEAN_10022604 | _ |
| Chr02:11030000-19249999 | BG_GLEAN_10022606 | _ |
| Chr02:11030000-19249999 | BG_GLEAN_10022607 | _ |
| Chr02:11030000-19249999 | BG_GLEAN_10022608 | \| Symbols: \| CONTAINS InterPro DOMAIN/s: Putative harbinger transposase-derived nuclease (InterPro:IPR006912); BEST Arabidopsis thaliana protein match is: unknown protein (TAIR:AT5G41980.1); Has 30201 Blast hits to 17322 proteins in 780 species: Archae - 12; Bacteria - 1396; Metazoa - 17338; Fungi - 3422; Plants - 5037; Viruses - 0; Other Eukaryotes - 2996 (source: NCBI BLink). \| chr5:13869120-13869941 FORWARD LENGTH=211 |
| Chr02:11030000-19249999 | BG_GLEAN_10022609 | _ |
| Chr02:11030000-19249999 | BG_GLEAN_10022610 | \| Symbols: \| nucleolar essential protein-related \| chr3:21092610-21094109 FORWARD LENGTH=298 |
| Chr02:11030000-19249999 | BG_GLEAN_10022611 | \| Symbols: ORF158 \| DNA/RNA polymerases superfamily protein \| chrM:235916-236392 FORWARD LENGTH=158 |
| Chr02:11030000-19249999 | BG_GLEAN_10022612 | _ |
| Chr02:11030000-19249999 | BG_GLEAN_10022614 | \| Symbols: \| CONTAINS InterPro DOMAIN/s: Retrotransposon gag protein (InterPro:IPR005162); Has 707 Blast hits to 705 proteins in 25 species: Archae - 0; Bacteria - 0; Metazoa - 4; Fungi - 0; Plants - 703; Viruses - 0; Other Eukaryotes - 0 (source: NCBI BLink). \| chr1:7447690-7448403 REVERSE LENGTH=237 |
| Chr02:11030000-19249999 | BG_GLEAN_10022615 | _ |
| Chr02:11030000-19249999 | BG_GLEAN_10022616 | \| Symbols: AGD4 \| ARF-GAP domain 4 \| chr1:3616905-3623612 REVERSE LENGTH=775 |
| Chr02:11030000-19249999 | BG_GLEAN_10022618 | \| Symbols: CRK8 \| cysteine-rich RLK (RECEPTOR-like protein kinase) 8 \| chr4:12129485-12134086 FORWARD LENGTH=1262 |
| Chr02:11030000-19249999 | BG_GLEAN_10022619 | \| Symbols: ORF158 \| DNA/RNA polymerases superfamily protein \| chrM:235916-236392 FORWARD LENGTH=158 |
| Chr02:11030000-19249999 | BG_GLEAN_10022620 | _ |
| Chr02:11030000-19249999 | BG_GLEAN_10022621 | _ |
| Chr02:11030000-19249999 | BG_GLEAN_10022623 | _ |
| Chr02:11030000-19249999 | BG_GLEAN_10022624 | _ |
| Chr02:11030000-19249999 | BG_GLEAN_10022625 | \| Symbols: ATGA2OX8, GA2OX8 \| gibberellin 2-oxidase 8 \| chr4:11302751-11306601 FORWARD LENGTH=338 |
| Chr02:11030000-19249999 | BG_GLEAN_10022626 | _ |
| Chr02:11030000-19249999 | BG_GLEAN_10022627 | \| Symbols: SPH1 \| S-protein homologue 1 \| chr4:9215680-9216135 REVERSE LENGTH=151 |
| Chr02:11030000-19249999 | BG_GLEAN_10022628 | _ |
| Chr02:11030000-19249999 | BG_GLEAN_10022629 | _ |
| Chr02:11030000-19249999 | BG_GLEAN_10022630 | _ |
| Chr02:11030000-19249999 | BG_GLEAN_10022631 | _ |
| Chr02:11030000-19249999 | BG_GLEAN_10022632 | _ |
| Chr02:11030000-19249999 | BG_GLEAN_10022633 | \| Symbols: ORF145A \| Gag-Pol-related retrotransposon family protein \| chrM:89617-90054 REVERSE LENGTH=145 |
| Chr02:11030000-19249999 | BG_GLEAN_10022634 | \| Symbols: CRK8 \| cysteine-rich RLK (RECEPTOR-like protein kinase) 8 \| chr4:12129485-12134086 FORWARD LENGTH=1262 |
| Chr02:11030000-19249999 | BG_GLEAN_10022635 | \| Symbols: At17.1 \| Encodes a protein whose expression is responsive to nematode infection. \| chr2:164014-165068 FORWARD LENGTH=215 |
| Chr02:11030000-19249999 | BG_GLEAN_10022637 | \| Symbols: \| ARM repeat superfamily protein \| chr1:26790825-26793105 REVERSE LENGTH=628 |
| Chr02:11030000-19249999 | BG_GLEAN_10022642 | \| Symbols: ANNAT5, ANN5 \| annexin 5 \| chr1:25519442-25520774 REVERSE LENGTH=316 |
| Chr02:11030000-19249999 | BG_GLEAN_10022643 | \| Symbols: ANNAT5, ANN5 \| annexin 5 \| chr1:25519442-25520774 REVERSE LENGTH=316 |
| Chr02:11030000-19249999 | BG_GLEAN_10022647 | _ |
| Chr02:11030000-19249999 | BG_GLEAN_10022648 | _ |
| Chr02:11030000-19249999 | BG_GLEAN_10022651 | _ |
| Chr02:11030000-19249999 | BG_GLEAN_10022652 | \| Symbols: \| zinc knuckle (CCHC-type) family protein \| chr4:422732-424580 REVERSE LENGTH=488 |
| Chr02:11030000-19249999 | BG_GLEAN_10022653 | \| Symbols: CRK8 \| cysteine-rich RLK (RECEPTOR-like protein kinase) 8 \| chr4:12129485-12134086 FORWARD LENGTH=1262 |
| Chr02:11030000-19249999 | BG_GLEAN_10022654 | \| Symbols: FAB1C \| FORMS APLOID AND BINUCLEATE CELLS 1C \| chr1:26782839-26788712 FORWARD LENGTH=1648 |
| Chr02:11030000-19249999 | BG_GLEAN_10022655 | \| Symbols: QPT \| quinolinate phoshoribosyltransferase \| chr2:165332-166842 REVERSE LENGTH=281 |
| Chr02:11030000-19249999 | BG_GLEAN_10022656 | _ |
| Chr02:11030000-19249999 | BG_GLEAN_10022657 | \| Symbols: QPT \| quinolinate phoshoribosyltransferase \| chr2:165332-167058 REVERSE LENGTH=327 |
| Chr02:11030000-19249999 | BG_GLEAN_10022658 | \| Symbols: \| NHL domain-containing protein \| chr2:175209-176372 REVERSE LENGTH=387 |
| Chr02:11030000-19249999 | BG_GLEAN_10022659 | _ |
| Chr02:11030000-19249999 | BG_GLEAN_10022660 | _ |
| Chr02:11030000-19249999 | BG_GLEAN_10022662 | \| Symbols: \| unknown protein; BEST Arabidopsis thaliana protein match is: unknown protein (TAIR:AT4G07350.1); Has 91 Blast hits to 77 proteins in 25 species: Archae - 0; Bacteria - 6; Metazoa - 5; Fungi - 5; Plants - 59; Viruses - 0; Other Eukaryotes - 16 (source: NCBI BLink). \| chr1:15240553-15244036 FORWARD LENGTH=567 |
| Chr02:11030000-19249999 | BG_GLEAN_10022663 | _ |
| Chr02:11030000-19249999 | BG_GLEAN_10022665 | _ |
| Chr02:11030000-19249999 | BG_GLEAN_10022666 | _ |
| Chr02:11030000-19249999 | BG_GLEAN_10022667 | _ |
| Chr02:11030000-19249999 | BG_GLEAN_10022668 | \| Symbols: CRK8 \| cysteine-rich RLK (RECEPTOR-like protein kinase) 8 \| chr4:12129485-12134086 FORWARD LENGTH=1262 |
| Chr02:11030000-19249999 | BG_GLEAN_10022669 | \| Symbols: CRK8 \| cysteine-rich RLK (RECEPTOR-like protein kinase) 8 \| chr4:12129485-12134086 FORWARD LENGTH=1262 |
| Chr02:11030000-19249999 | BG_GLEAN_10022671 | \| Symbols: \| hydroxyproline-rich glycoprotein family protein \| chr1:8165025-8165459 FORWARD LENGTH=144 |
| Chr02:11030000-19249999 | BG_GLEAN_10022672 | _ |
| Chr02:11030000-19249999 | BG_GLEAN_10022674 | _ |
| Chr02:11030000-19249999 | BG_GLEAN_10022675 | \| Symbols: \| unknown protein; Has 68 Blast hits to 67 proteins in 12 species: Archae - 0; Bacteria - 0; Metazoa - 0; Fungi - 0; Plants - 68; Viruses - 0; Other Eukaryotes - 0 (source: NCBI BLink). \| chr4:7753436-7754086 FORWARD LENGTH=216 |
| Chr02:11030000-19249999 | BG_GLEAN_10022676 | _ |
| Chr02:11030000-19249999 | BG_GLEAN_10022677 | _ |
| Chr02:11030000-19249999 | BG_GLEAN_10022679 | \| Symbols: \| unknown protein; FUNCTIONS IN: molecular_function unknown; INVOLVED IN: biological_process unknown; LOCATED IN: cellular_component unknown; EXPRESSED IN: egg cell; Has 84 Blast hits to 81 proteins in 31 species: Archae - 0; Bacteria - 0; Metazoa - 42; Fungi - 0; Plants - 41; Viruses - 0; Other Eukaryotes - 1 (source: NCBI BLink). \| chr5:2332527-2335736 FORWARD LENGTH=642 |
| Chr02:11030000-19249999 | BG_GLEAN_10022680 | \| Symbols: PIN7, ATPIN7 \| Auxin efflux carrier family protein \| chr1:8180768-8183406 REVERSE LENGTH=619 |
| Chr02:11030000-19249999 | BG_GLEAN_10022682 | \| Symbols: \| zinc knuckle (CCHC-type) family protein \| chr4:422732-424580 REVERSE LENGTH=488 |
| Chr02:11030000-19249999 | BG_GLEAN_10022683 | \| Symbols: SPH1 \| S-protein homologue 1 \| chr4:9215680-9216135 REVERSE LENGTH=151 |
| Chr02:11030000-19249999 | BG_GLEAN_10022684 | _ |
| Chr02:11030000-19249999 | BG_GLEAN_10022686 | \| Symbols: ORF158 \| DNA/RNA polymerases superfamily protein \| chrM:235916-236392 FORWARD LENGTH=158 |
| Chr02:11030000-19249999 | BG_GLEAN_10022688 | _ |
| Chr02:11030000-19249999 | BG_GLEAN_10022689 | _ |
| Chr02:11030000-19249999 | BG_GLEAN_10022690 | _ |
| Chr02:11030000-19249999 | BG_GLEAN_10022691 | _ |
| Chr02:11030000-19249999 | BG_GLEAN_10022693 | \| Symbols: SPH1 \| S-protein homologue 1 \| chr4:9215680-9216135 REVERSE LENGTH=151 |
| Chr02:11030000-19249999 | BG_GLEAN_10022694 | _ |
| Chr02:11030000-19249999 | BG_GLEAN_10022695 | \| Symbols: ORF145A \| Gag-Pol-related retrotransposon family protein \| chrM:89617-90054 REVERSE LENGTH=145 |
| Chr02:11030000-19249999 | BG_GLEAN_10022696 | \| Symbols: \| CONTAINS InterPro DOMAIN/s: Retrotransposon gag protein (InterPro:IPR005162); BEST Arabidopsis thaliana protein match is: unknown protein (TAIR:AT1G34070.1); Has 1807 Blast hits to 1807 proteins in 277 species: Archae - 0; Bacteria - 0; Metazoa - 736; Fungi - 347; Plants - 385; Viruses - 0; Other Eukaryotes - 339 (source: NCBI BLink). \| chr5:19472661-19473770 REVERSE LENGTH=369 |
| Chr02:11030000-19249999 | BG_GLEAN_10022697 | \| Symbols: ORF145A \| Gag-Pol-related retrotransposon family protein \| chrM:89617-90054 REVERSE LENGTH=145 |
| Chr02:11030000-19249999 | BG_GLEAN_10022698 | \| Symbols: \| CONTAINS InterPro DOMAIN/s: Retrotransposon gag protein (InterPro:IPR005162); BEST Arabidopsis thaliana protein match is: unknown protein (TAIR:AT1G34070.1); Has 1807 Blast hits to 1807 proteins in 277 species: Archae - 0; Bacteria - 0; Metazoa - 736; Fungi - 347; Plants - 385; Viruses - 0; Other Eukaryotes - 339 (source: NCBI BLink). \| chr5:19472661-19473770 REVERSE LENGTH=369 |
| Chr02:11030000-19249999 | BG_GLEAN_10022699 | \| Symbols: \| Protein of unknown function, DUF538 \| chr1:3009109-3009648 FORWARD LENGTH=179 |
| Chr02:11030000-19249999 | BG_GLEAN_10022700 | _ |
| Chr02:11030000-19249999 | BG_GLEAN_10022701 | _ |
| Chr02:11030000-19249999 | BG_GLEAN_10022702 | \| Symbols: ORF158 \| DNA/RNA polymerases superfamily protein \| chrM:235916-236392 FORWARD LENGTH=158 |
| Chr02:11030000-19249999 | BG_GLEAN_10022703 | \| Symbols: \| Zinc knuckle (CCHC-type) family protein \| chr2:6588743-6590167 REVERSE LENGTH=474 |
| Chr02:11030000-19249999 | BG_GLEAN_10022705 | \| Symbols: CRK8 \| cysteine-rich RLK (RECEPTOR-like protein kinase) 8 \| chr4:12129485-12134086 FORWARD LENGTH=1262 |
| Chr02:11030000-19249999 | BG_GLEAN_10022707 | _ |
| Chr02:11030000-19249999 | BG_GLEAN_10022708 | \| Symbols: \| Calcium-dependent lipid-binding (CaLB domain) family protein \| chr2:242297-243233 REVERSE LENGTH=180 |
| Chr02:11030000-19249999 | BG_GLEAN_10022709 | \| Symbols: \| Gag-Pol-related retrotransposon family protein \| chr3:7363921-7365138 FORWARD LENGTH=405 |
| Chr02:11030000-19249999 | BG_GLEAN_10022710 | \| Symbols: \| phosphoglucomutase, putative / glucose phosphomutase, putative \| chr1:26705594-26708034 FORWARD LENGTH=615 |
| Chr02:11030000-19249999 | BG_GLEAN_10022711 | \| Symbols: ORF158 \| DNA/RNA polymerases superfamily protein \| chrM:235916-236392 FORWARD LENGTH=158 |
| Chr02:11030000-19249999 | BG_GLEAN_10022712 | \| Symbols: emb1129 \| Nucleic acid-binding, OB-fold-like protein \| chr1:18283156-18283643 FORWARD LENGTH=116 |
| Chr02:11030000-19249999 | BG_GLEAN_10022713 | _ |
| Chr02:11030000-19249999 | BG_GLEAN_10022714 | _ |
| Chr02:11030000-19249999 | BG_GLEAN_10022715 | _ |
| Chr02:11030000-19249999 | BG_GLEAN_10022717 | \| Symbols: \| Protein of unknown function DUF2359, transmembrane \| chr1:26688622-26691185 REVERSE LENGTH=610 |
| Chr02:11030000-19249999 | BG_GLEAN_10022719 | \| Symbols: CRR3 \| chlororespiratory reduction 3 \| chr2:266675-267379 FORWARD LENGTH=174 |
| Chr02:11030000-19249999 | BG_GLEAN_10022720 | _ |
| Chr02:11030000-19249999 | BG_GLEAN_10022721 | \| Symbols: \| MuDR family transposase \| chr1:23847756-23849915 FORWARD LENGTH=719 |
| Chr02:11030000-19249999 | BG_GLEAN_10022722 | \| Symbols: \| Protein kinase superfamily protein \| chr1:26673847-26675687 REVERSE LENGTH=425 |
| Chr02:11030000-19249999 | BG_GLEAN_10022723 | \| Symbols: \| ENTH/ANTH/VHS superfamily protein \| chr2:268975-272356 FORWARD LENGTH=571 |
| Chr02:11030000-19249999 | BG_GLEAN_10022724 | _ |
| Chr02:11030000-19249999 | BG_GLEAN_10022725 | _ |
| Chr02:11030000-19249999 | BG_GLEAN_10022726 | _ |
| Chr02:11030000-19249999 | BG_GLEAN_10022727 | \| Symbols: \| Clathrin adaptor complex small chain family protein \| chr3:18902346-18903959 FORWARD LENGTH=166 |
| Chr02:11030000-19249999 | BG_GLEAN_10022728 | _ |
| Chr02:11030000-19249999 | BG_GLEAN_10022729 | _ |
| Chr02:11030000-19249999 | BG_GLEAN_10022730 | \| Symbols: \| PLAC8 family protein \| chr5:13707084-13707818 FORWARD LENGTH=152 |
| Chr02:11030000-19249999 | BG_GLEAN_10022731 | \| Symbols: \| O-Glycosyl hydrolases family 17 protein \| chr2:279541-281955 REVERSE LENGTH=501 |
| Chr02:11030000-19249999 | BG_GLEAN_10022732 | \| Symbols: \| O-Glycosyl hydrolases family 17 protein \| chr2:279541-281955 REVERSE LENGTH=501 |
| Chr02:11030000-19249999 | BG_GLEAN_10022733 | _ |
| Chr02:11030000-19249999 | BG_GLEAN_10022734 | \| Symbols: \| MuDR family transposase \| chr1:23847756-23849915 FORWARD LENGTH=719 |
| Chr02:11030000-19249999 | BG_GLEAN_10022735 | _ |
| Chr02:11030000-19249999 | BG_GLEAN_10022736 | _ |
| Chr02:11030000-19249999 | BG_GLEAN_10022738 | \| Symbols: CRK8 \| cysteine-rich RLK (RECEPTOR-like protein kinase) 8 \| chr4:12129485-12134086 FORWARD LENGTH=1262 |
| Chr02:11030000-19249999 | BG_GLEAN_10022739 | \| Symbols: JAZ3, JAI3, TIFY6B \| jasmonate-zim-domain protein 3 \| chr3:6119968-6122691 FORWARD LENGTH=352 |
| Chr02:11030000-19249999 | BG_GLEAN_10022740 | _ |
| Chr02:11030000-19249999 | BG_GLEAN_10022741 | _ |
| Chr02:11030000-19249999 | BG_GLEAN_10022742 | _ |
| Chr02:11030000-19249999 | BG_GLEAN_10022743 | _ |
| Chr02:11030000-19249999 | BG_GLEAN_10022744 | \| Symbols: \| FUNCTIONS IN: molecular_function unknown; INVOLVED IN: intracellular protein transport; LOCATED IN: endomembrane system, integral to membrane, endoplasmic reticulum; EXPRESSED IN: 23 plant structures; EXPRESSED DURING: 13 growth stages; CONTAINS InterPro DOMAIN/s: B-cell receptor-associated 31-like (InterPro:IPR008417); BEST Arabidopsis thaliana protein match is: unknown protein (TAIR:AT3G03160.1); Has 1807 Blast hits to 1807 proteins in 277 species: Archae - 0; Bacteria - 0; Metazoa - 736; Fungi - 347; Plants - 385; Viruses - 0; Other Eukaryotes - 339 (source: NCBI BLink). \| chr5:5652310-5652702 FORWARD LENGTH=130 |
| Chr02:11030000-19249999 | BG_GLEAN_10022745 | _ |
| Chr02:11030000-19249999 | BG_GLEAN_10022746 | _ |
| Chr02:11030000-19249999 | BG_GLEAN_10022747 | _ |
| Chr02:11030000-19249999 | BG_GLEAN_10022748 | _ |
| Chr02:11030000-19249999 | BG_GLEAN_10022750 | \| Symbols: PDLP6 \| plasmodesmata-located protein 6 \| chr2:291717-292496 REVERSE LENGTH=259 |
| Chr02:11030000-19249999 | BG_GLEAN_10022751 | \| Symbols: \| Caleosin-related family protein \| chr1:26644830-26645970 FORWARD LENGTH=195 |
| Chr02:11030000-19249999 | BG_GLEAN_10022753 | \| Symbols: MMZ1, UEV1A \| MMS ZWEI homologue 1 \| chr1:8257209-8258573 REVERSE LENGTH=158 |
| Chr02:11030000-19249999 | BG_GLEAN_10022754 | _ |
| Chr02:11030000-19249999 | BG_GLEAN_10022755 | _ |
| Chr02:11030000-19249999 | BG_GLEAN_10022757 | _ |
| Chr02:11030000-19249999 | BG_GLEAN_10022758 | \| Symbols: \| 2-oxoglutarate (2OG) and Fe(II)-dependent oxygenase superfamily protein \| chr1:2035909-2037186 FORWARD LENGTH=369 |
| Chr02:11030000-19249999 | BG_GLEAN_10022759 | \| Symbols: \| EF hand calcium-binding protein family \| chr1:8723893-8724453 REVERSE LENGTH=186 |
| Chr02:11030000-19249999 | BG_GLEAN_10022760 | \| Symbols: \| ARM repeat superfamily protein \| chr2:309144-313499 REVERSE LENGTH=743 |
| Chr02:11030000-19249999 | BG_GLEAN_10022761 | \| Symbols: \| ARM repeat superfamily protein \| chr2:309144-313499 REVERSE LENGTH=743 |
| Chr02:11030000-19249999 | BG_GLEAN_10022763 | _ |
| Chr02:11030000-19249999 | BG_GLEAN_10022765 | \| Symbols: NUP155 \| nucleoporin 155 \| chr1:5116921-5123259 REVERSE LENGTH=1464 |
| Chr02:11030000-19249999 | BG_GLEAN_10022766 | _ |
| Chr02:11030000-19249999 | BG_GLEAN_10022767 | \| Symbols: EMB1895 \| ARM repeat superfamily protein \| chr4:10854790-10859330 REVERSE LENGTH=1134 |
| Chr02:11030000-19249999 | BG_GLEAN_10022768 | \| Symbols: ORF120 \| Polynucleotidyl transferase, ribonuclease H-like superfamily protein \| chrM:207553-207915 REVERSE LENGTH=120 |
| Chr02:11030000-19249999 | BG_GLEAN_10022769 | _ |
| Chr02:11030000-19249999 | BG_GLEAN_10022770 | _ |
| Chr02:11030000-19249999 | BG_GLEAN_10022772 | \| Symbols: \| BED zinc finger ;hAT family dimerisation domain \| chr3:14321838-14323928 FORWARD LENGTH=696 |
| Chr02:11030000-19249999 | BG_GLEAN_10022773 | \| Symbols: CRK8 \| cysteine-rich RLK (RECEPTOR-like protein kinase) 8 \| chr4:12129485-12134086 FORWARD LENGTH=1262 |
| Chr02:11030000-19249999 | BG_GLEAN_10022775 | \| Symbols: \| UDP-3-O-acyl N-acetylglycosamine deacetylase family protein \| chr1:8767581-8769207 FORWARD LENGTH=326 |
| Chr02:11030000-19249999 | BG_GLEAN_10022776 | \| Symbols: \| RNA-directed DNA polymerase (reverse transcriptase)-related family protein \| chr1:16508723-16509784 REVERSE LENGTH=320 |
| Chr02:11030000-19249999 | BG_GLEAN_10022777 | \| Symbols: \| DNAse I-like superfamily protein \| chr1:16528880-16531065 REVERSE LENGTH=626 |
| Chr02:11030000-19249999 | BG_GLEAN_10022778 | _ |
| Chr02:11030000-19249999 | BG_GLEAN_10022779 | _ |
| Chr02:11030000-19249999 | BG_GLEAN_10022780 | _ |
| Chr02:11030000-19249999 | BG_GLEAN_10022781 | _ |
| Chr02:11030000-19249999 | BG_GLEAN_10022782 | _ |
| Chr02:11030000-19249999 | BG_GLEAN_10022783 | _ |
| Chr02:11030000-19249999 | BG_GLEAN_10022784 | _ |
| Chr02:11030000-19249999 | BG_GLEAN_10022785 | _ |
| Chr02:11030000-19249999 | BG_GLEAN_10022786 | \| Symbols: \| aminoacyl-tRNA ligases;ATP binding;nucleotide binding \| chr3:14838392-14841189 REVERSE LENGTH=818 |
| Chr02:11030000-19249999 | BG_GLEAN_10022787 | \| Symbols: ATMAP70-2, MAP70-2 \| microtubule-associated proteins 70-2 \| chr1:8760001-8763256 REVERSE LENGTH=634 |
| Chr02:11030000-19249999 | BG_GLEAN_10022788 | _ |
| Chr02:11030000-19249999 | BG_GLEAN_10022789 | \| Symbols: SPH1 \| S-protein homologue 1 \| chr4:9215680-9216135 REVERSE LENGTH=151 |
| Chr02:11030000-19249999 | BG_GLEAN_10022790 | _ |
| Chr02:11030000-19249999 | BG_GLEAN_10022792 | _ |
| Chr02:11030000-19249999 | BG_GLEAN_10022797 | \| Symbols: \| Plant self-incompatibility protein S1 family \| chr4:12843802-12844209 FORWARD LENGTH=135 |
| Chr02:11030000-19249999 | BG_GLEAN_10022798 | \| Symbols: \| Plant self-incompatibility protein S1 family \| chr4:12843802-12844209 FORWARD LENGTH=135 |
| Chr02:11030000-19249999 | BG_GLEAN_10022799 | _ |
| Chr02:11030000-19249999 | BG_GLEAN_10022800 | \| Symbols: CRK8 \| cysteine-rich RLK (RECEPTOR-like protein kinase) 8 \| chr4:12129485-12134086 FORWARD LENGTH=1262 |
| Chr02:11030000-19249999 | BG_GLEAN_10022801 | \| Symbols: \| Plant self-incompatibility protein S1 family \| chr4:12843802-12844209 FORWARD LENGTH=135 |
| Chr02:11030000-19249999 | BG_GLEAN_10022803 | _ |
| Chr02:11030000-19249999 | BG_GLEAN_10022805 | _ |
| Chr02:11030000-19249999 | BG_GLEAN_10022806 | _ |
| Chr02:11030000-19249999 | BG_GLEAN_10022807 | _ |
| Chr02:11030000-19249999 | BG_GLEAN_10022808 | \| Symbols: \| Glycosyltransferase family 61 protein \| chr2:17360654-17362391 FORWARD LENGTH=500 |
| Chr02:11030000-19249999 | BG_GLEAN_10022809 | \| Symbols: \| Pentatricopeptide repeat (PPR) superfamily protein \| chr3:4949385-4951346 REVERSE LENGTH=653 |
| Chr02:11030000-19249999 | BG_GLEAN_10022810 | \| Symbols: \| Cyclin-like family protein \| chr3:3199907-3201642 FORWARD LENGTH=312 |
| Chr02:11030000-19249999 | BG_GLEAN_10022812 | _ |
| Chr02:11030000-19249999 | BG_GLEAN_10022813 | _ |
| Chr02:11030000-19249999 | BG_GLEAN_10022814 | _ |
| Chr02:11030000-19249999 | BG_GLEAN_10022815 | _ |
| Chr02:11030000-19249999 | BG_GLEAN_10022816 | _ |
| Chr02:11030000-19249999 | BG_GLEAN_10022817 | _ |
| Chr02:11030000-19249999 | BG_GLEAN_10022819 | _ |
| Chr02:11030000-19249999 | BG_GLEAN_10022820 | \| Symbols: UGT74D1 \| UDP-glucosyl transferase 74D1 \| chr2:13497312-13499870 FORWARD LENGTH=456 |
| Chr02:11030000-19249999 | BG_GLEAN_10022823 | _ |
| Chr02:11030000-19249999 | BG_GLEAN_10022824 | _ |
| Chr02:11030000-19249999 | BG_GLEAN_10022825 | _ |
| Chr02:11030000-19249999 | BG_GLEAN_10022827 | \| Symbols: UGT72E1 \| UDP-glucosyl transferase 72E1 \| chr3:18855348-18856811 REVERSE LENGTH=487 |
| Chr02:11030000-19249999 | BG_GLEAN_10022828 | _ |
| Chr02:11030000-19249999 | BG_GLEAN_10022832 | _ |
| Chr02:11030000-19249999 | BG_GLEAN_10022833 | _ |
| Chr02:11030000-19249999 | BG_GLEAN_10022834 | _ |
| Chr02:11030000-19249999 | BG_GLEAN_10022836 | \| Symbols: UGT73B2 \| UDP-glucosyltransferase 73B2 \| chr4:16345476-16347016 REVERSE LENGTH=483 |
| Chr02:11030000-19249999 | BG_GLEAN_10022837 | _ |
| Chr02:11030000-19249999 | BG_GLEAN_10022841 | _ |
| Chr02:11030000-19249999 | BG_GLEAN_10022842 | \| Symbols: \| ATP binding microtubule motor family protein \| chr3:23441065-23443809 REVERSE LENGTH=465 |
