# Supplementary material for: QTL-Seq identifies quantitative trait loci of relative electrical conductivity associated with heat tolerance in bottle gourd (Lagenaria siceraria)
Source: PLoS One. 2020 Nov 10;15(11):e0227663. doi: 10.1371/journal.pone.0227663 (PMC7654804; doi:10.1371/journal.pone.0227663)
Supplement: S2 Table — (DOCX) [file pone.0227663.s005.docx]

**S2 TABLE** Details of 62 nonsynonymous and stoploss type of SNPs in the *qHT2.1* region

| Chr | type | Gene | detail | function |
| --- | --- | --- | --- | --- |
| Chr02:11030000-19249999 | nonsynonymous | BG_GLEAN_10022276 | T1115C:L372P | _ |
| Chr02:11030000-19249999 | nonsynonymous | BG_GLEAN_10022339 | G511A:V171I | \| Symbols: PQL1, PQL2 \| PsbQ-like 1 \| chr3:168478-169407 FORWARD LENGTH=220 |
| Chr02:11030000-19249999 | nonsynonymous | BG_GLEAN_10022346 | G1018A:G340R | \| Symbols: ORF158 \| DNA/RNA polymerases superfamily protein \| chrM:235916-236392 FORWARD LENGTH=158 |
| Chr02:11030000-19249999 | nonsynonymous | BG_GLEAN_10022369 | C713A:A238D | \| Symbols: ATATG18F, ATG18F, G18F \| homolog of yeast autophagy 18 (ATG18) F \| chr5:22233977-22236804 REVERSE LENGTH=763 |
| Chr02:11030000-19249999 | stopgain | BG_GLEAN_10022402 | G335A:W112X | _ |
| Chr02:11030000-19249999 | nonsynonymous | BG_GLEAN_10022457 | A341G:E114G | \| Symbols: CRK8 \| cysteine-rich RLK (RECEPTOR-like protein kinase) 8 \| chr4:12129485-12134086 FORWARD LENGTH=1262 |
| Chr02:11030000-19249999 | nonsynonymous | BG_GLEAN_10022458 | T707C:V236A | \| Symbols: CRK8 \| cysteine-rich RLK (RECEPTOR-like protein kinase) 8 \| chr4:12129485-12134086 FORWARD LENGTH=1262 |
| Chr02:11030000-19249999 | stoploss | BG_GLEAN_10022459 | T670C:X224R | \| Symbols: \| CONTAINS InterPro DOMAIN/s: Retrotransposon gag protein (InterPro:IPR005162); Has 707 Blast hits to 705 proteins in 25 species: Archae - 0; Bacteria - 0; Metazoa - 4; Fungi - 0; Plants - 703; Viruses - 0; Other Eukaryotes - 0 (source: NCBI BLink). \| chr1:7447690-7448403 REVERSE LENGTH=237 |
| Chr02:11030000-19249999 | nonsynonymous | BG_GLEAN_10022459 | A655C:N219H | \| Symbols: \| CONTAINS InterPro DOMAIN/s: Retrotransposon gag protein (InterPro:IPR005162); Has 707 Blast hits to 705 proteins in 25 species: Archae - 0; Bacteria - 0; Metazoa - 4; Fungi - 0; Plants - 703; Viruses - 0; Other Eukaryotes - 0 (source: NCBI BLink). \| chr1:7447690-7448403 REVERSE LENGTH=237 |
| Chr02:11030000-19249999 | nonsynonymous | BG_GLEAN_10022500 | T422C:I141T | _ |
| Chr02:11030000-19249999 | nonsynonymous | BG_GLEAN_10022502 | A1183T:I395F | \| Symbols: ATSBT1.1, SBTI1.1 \| subtilase family protein \| chr1:310332-313011 FORWARD LENGTH=774 |
| Chr02:11030000-19249999 | nonsynonymous | BG_GLEAN_10022504 | C38T:T13I | _ |
| Chr02:11030000-19249999 | nonsynonymous | BG_GLEAN_10022508 | C119T:S40L | _ |
| Chr02:11030000-19249999 | nonsynonymous | BG_GLEAN_10022511 | G463T:D155Y | \| Symbols: \| DEAD/DEAH box RNA helicase family protein \| chr2:193950-199056 REVERSE LENGTH=973 |
| Chr02:11030000-19249999 | nonsynonymous | BG_GLEAN_10022513 | C155G:T52S | \| Symbols: BGAL7 \| beta-galactosidase 7 \| chr5:7010536-7013994 FORWARD LENGTH=826 |
| Chr02:11030000-19249999 | nonsynonymous | BG_GLEAN_10022513 | G390C:W130C | \| Symbols: BGAL7 \| beta-galactosidase 7 \| chr5:7010536-7013994 FORWARD LENGTH=826 |
| Chr02:11030000-19249999 | nonsynonymous | BG_GLEAN_10022513 | T395A:L132H | \| Symbols: BGAL7 \| beta-galactosidase 7 \| chr5:7010536-7013994 FORWARD LENGTH=826 |
| Chr02:11030000-19249999 | nonsynonymous | BG_GLEAN_10022514 | G1007A:S336N | \| Symbols: BGAL7 \| beta-galactosidase 7 \| chr5:7010536-7013994 FORWARD LENGTH=826 |
| Chr02:11030000-19249999 | nonsynonymous | BG_GLEAN_10022514 | G1010A:C337Y | \| Symbols: BGAL7 \| beta-galactosidase 7 \| chr5:7010536-7013994 FORWARD LENGTH=826 |
| Chr02:11030000-19249999 | nonsynonymous | BG_GLEAN_10022514 | T1043A:M348K | \| Symbols: BGAL7 \| beta-galactosidase 7 \| chr5:7010536-7013994 FORWARD LENGTH=826 |
| Chr02:11030000-19249999 | nonsynonymous | BG_GLEAN_10022515 | T80C:L27S | _ |
| Chr02:11030000-19249999 | stopgain | BG_GLEAN_10022515 | C91T:R31X | _ |
| Chr02:11030000-19249999 | nonsynonymous | BG_GLEAN_10022517 | A21G:I7M | _ |
| Chr02:11030000-19249999 | nonsynonymous | BG_GLEAN_10022517 | G471A:M157I | _ |
| Chr02:11030000-19249999 | nonsynonymous | BG_GLEAN_10022517 | A481G:R161G | _ |
| Chr02:11030000-19249999 | nonsynonymous | BG_GLEAN_10022530 | A329G:H110R | \| Symbols: ORF158 \| DNA/RNA polymerases superfamily protein \| chrM:235916-236392 FORWARD LENGTH=158 |
| Chr02:11030000-19249999 | nonsynonymous | BG_GLEAN_10022531 | C314T:A105V | _ |
| Chr02:11030000-19249999 | nonsynonymous | BG_GLEAN_10022533 | G676A:V226I | \| Symbols: CRK8 \| cysteine-rich RLK (RECEPTOR-like protein kinase) 8 \| chr4:12129485-12134086 FORWARD LENGTH=1262 |
| Chr02:11030000-19249999 | nonsynonymous | BG_GLEAN_10022534 | T2450C:I817T | \| Symbols: CRK8 \| cysteine-rich RLK (RECEPTOR-like protein kinase) 8 \| chr4:12129485-12134086 FORWARD LENGTH=1262 |
| Chr02:11030000-19249999 | nonsynonymous | BG_GLEAN_10022537 | C809T:T270I | _ |
| Chr02:11030000-19249999 | nonsynonymous | BG_GLEAN_10022538 | G476A:R159Q | _ |
| Chr02:11030000-19249999 | stopgain | BG_GLEAN_10022538 | C487T:Q163X | _ |
| Chr02:11030000-19249999 | nonsynonymous | BG_GLEAN_10022550 | C212G:A71G | \| Symbols: \| unknown protein; FUNCTIONS IN: molecular_function unknown; INVOLVED IN: biological_process unknown; LOCATED IN: endomembrane system; EXPRESSED IN: 22 plant structures; EXPRESSED DURING: 13 growth stages. \| chr1:5167354-5168151 REVERSE LENGTH=132 |
| Chr02:11030000-19249999 | nonsynonymous | BG_GLEAN_10022553 | G145A:G49R | _ |
| Chr02:11030000-19249999 | nonsynonymous | BG_GLEAN_10022553 | G220A:V74I | _ |
| Chr02:11030000-19249999 | nonsynonymous | BG_GLEAN_10022555 | C49T:R17W | \| Symbols: ORF158 \| DNA/RNA polymerases superfamily protein \| chrM:235916-236392 FORWARD LENGTH=158 |
| Chr02:11030000-19249999 | nonsynonymous | BG_GLEAN_10022556 | T1345C:S449P | \| Symbols: CRK8 \| cysteine-rich RLK (RECEPTOR-like protein kinase) 8 \| chr4:12129485-12134086 FORWARD LENGTH=1262 |
| Chr02:11030000-19249999 | nonsynonymous | BG_GLEAN_10022559 | G403A:A135T | _ |
| Chr02:11030000-19249999 | nonsynonymous | BG_GLEAN_10022559 | T754C:Y252H | _ |
| Chr02:11030000-19249999 | nonsynonymous | BG_GLEAN_10022560 | A1C:M1L | _ |
| Chr02:11030000-19249999 | stopgain | BG_GLEAN_10022562 | C217T:Q73X | _ |
| Chr02:11030000-19249999 | nonsynonymous | BG_GLEAN_10022562 | G15C:K5N | _ |
| Chr02:11030000-19249999 | nonsynonymous | BG_GLEAN_10022589 | A292G:K98E | _ |
| Chr02:11030000-19249999 | nonsynonymous | BG_GLEAN_10022591 | G223A:G75S | _ |
| Chr02:11030000-19249999 | nonsynonymous | BG_GLEAN_10022642 | C605A:S202Y | \| Symbols: ANNAT5, ANN5 \| annexin 5 \| chr1:25519442-25520774 REVERSE LENGTH=316 |
| Chr02:11030000-19249999 | nonsynonymous | BG_GLEAN_10022652 | C688G:R230G | \| Symbols: \| zinc knuckle (CCHC-type) family protein \| chr4:422732-424580 REVERSE LENGTH=488 |
| Chr02:11030000-19249999 | nonsynonymous | BG_GLEAN_10022653 | A193G:K65E | \| Symbols: CRK8 \| cysteine-rich RLK (RECEPTOR-like protein kinase) 8 \| chr4:12129485-12134086 FORWARD LENGTH=1262 |
| Chr02:11030000-19249999 | nonsynonymous | BG_GLEAN_10022653 | C508T:L170F | \| Symbols: CRK8 \| cysteine-rich RLK (RECEPTOR-like protein kinase) 8 \| chr4:12129485-12134086 FORWARD LENGTH=1262 |
| Chr02:11030000-19249999 | nonsynonymous | BG_GLEAN_10022679 | G1574A:G525D | \| Symbols: \| unknown protein; FUNCTIONS IN: molecular_function unknown; INVOLVED IN: biological_process unknown; LOCATED IN: cellular_component unknown; EXPRESSED IN: egg cell; Has 84 Blast hits to 81 proteins in 31 species: Archae - 0; Bacteria - 0; Metazoa - 42; Fungi - 0; Plants - 41; Viruses - 0; Other Eukaryotes - 1 (source: NCBI BLink). \| chr5:2332527-2335736 FORWARD LENGTH=642 |
| Chr02:11030000-19249999 | nonsynonymous | BG_GLEAN_10022713 | A230G:K77R | _ |
| Chr02:11030000-19249999 | stopgain | BG_GLEAN_10022720 | C25T:Q9X | _ |
| Chr02:11030000-19249999 | nonsynonymous | BG_GLEAN_10022727 | T118A:C40S | \| Symbols: \| Clathrin adaptor complex small chain family protein \| chr3:18902346-18903959 FORWARD LENGTH=166 |
| Chr02:11030000-19249999 | nonsynonymous | BG_GLEAN_10022734 | A616G:I206V | \| Symbols: \| MuDR family transposase \| chr1:23847756-23849915 FORWARD LENGTH=719 |
| Chr02:11030000-19249999 | nonsynonymous | BG_GLEAN_10022735 | G130A:V44I | _ |
| Chr02:11030000-19249999 | nonsynonymous | BG_GLEAN_10022766 | A121G:N41D | _ |
| Chr02:11030000-19249999 | nonsynonymous | BG_GLEAN_10022767 | G1076A:R359Q | \| Symbols: EMB1895 \| ARM repeat superfamily protein \| chr4:10854790-10859330 REVERSE LENGTH=1134 |
| Chr02:11030000-19249999 | stopgain | BG_GLEAN_10022769 | C239G:S80X | _ |
| Chr02:11030000-19249999 | nonsynonymous | BG_GLEAN_10022770 | G1162A:G388R | _ |
| Chr02:11030000-19249999 | nonsynonymous | BG_GLEAN_10022776 | A65G:E22G | \| Symbols: \| RNA-directed DNA polymerase (reverse transcriptase)-related family protein \| chr1:16508723-16509784 REVERSE LENGTH=320 |
| Chr02:11030000-19249999 | nonsynonymous | BG_GLEAN_10022777 | A37G:S13G | \| Symbols: \| DNAse I-like superfamily protein \| chr1:16528880-16531065 REVERSE LENGTH=626 |
| Chr02:11030000-19249999 | stopgain | BG_GLEAN_10022779 | C205T:R69X | _ |
| Chr02:11030000-19249999 | nonsynonymous | BG_GLEAN_10022780 | C334T:P112S | _ |
