# Supplementary material for: QTL-Seq identifies quantitative trait loci of relative electrical conductivity associated with heat tolerance in bottle gourd (Lagenaria siceraria)
Source: PLoS One. 2020 Nov 10;15(11):e0227663. doi: 10.1371/journal.pone.0227663 (PMC7654804; doi:10.1371/journal.pone.0227663)
Supplement: S3 Table — (DOCX) [file pone.0227663.s006.docx]

**S3 TABLE** Function annotation of candidate genes

| Code | Gene | Start | End | Gene annotation | GO results | Gene function | Reference | Classification |
| --- | --- | --- | --- | --- | --- | --- | --- | --- |
| 1 | BG_GLEAN_10022767 | 18202876 | 18208313 | ARM repeat superfamily protein |  | gametogenesis | [67] | Pollen and flower sterility |
| 2 | BG_GLEAN_10022642 | 16351903 | 16356748 | annexin 5 | pollen tube growth  calcium-dependent | pollen development | [68] |  |
| 3 | BG_GLEAN_10022513 | 14863003 | 14864202 | beta-galactosidase 7 |  | pollen development | [69] |  |
| 4 | BG_GLEAN_10022514 | 14864272 | 14865477 |  |  |  |  |  |
| 5 | BG_GLEAN_10022346 | 12947058 | 12948329 | ORF158 | metal ion binding | Ogura cytoplasmic male sterility | [70] |  |
| 6 | BG_GLEAN_10022530 | 15056845 | 15057528 |  |  |  |  |  |
| 7 | BG_GLEAN_10022457 | 14198082 | 14198549 | CRK8 | cellular metabolic process | protect against oxidative stress | [73] | Defying oxidative and signal recognition |
| 8 | BG_GLEAN_10022458 | 14202592 | 14203758 |  |  |  |  |  |
|  | BG_GLEAN_10022533 | 15143557 | 15144330 |  |  |  |  |  |
| 10 | BG_GLEAN_10022534 | 15150418 | 15153009 |  |  |  |  |  |
| 11 | BG_GLEAN_10022556 | 15364584 | 15366248 |  |  |  |  |  |
| 12 | BG_GLEAN_10022653 | 16479728 | 16480867 |  |  |  |  |  |
| 13 | BG_GLEAN_10022511 | 14855903 | 14856478 | DEAD/DEAH box RNA helicase family protein |  | perform essential roles in stress response | [74] |  |
| 14 | BG_GLEAN_10022502 | 14706104 | 14708853 | serine-type peptidase activity |  | thermal tolerance | [75] |  |
| 15 | BG_GLEAN_10022339 | 12878315 | 12879195 | PQL1, PQL2 | single-organism carbohydrate metabolic process | photosystem II oxygen evolving complex/calcium ion binding | [76] |  |
| 16 | BG_GLEAN_10022589 | 15709012 | 15709407 | serine/threonine-protein kinase SRK2C-like (*Cucumissativus*) |  | fungus infection signal recognition and transduction | [84] |  |
| 17 | BG_GLEAN_10022369 | 13153956 | 13158125 | ATG18 | single-organism cellular process/cytoplasmic part | autophagy-related generesponses to stress | [85] | Autophagy |
| 18 | BG_GLEAN_10022459 | 14206108 | 14206779 | Retrotransposon gag protein |  | retrotransposon | [77] | Transcription factor |
| 19 | BG_GLEAN_10022500 | 14679103 | 14679579 |  |  |  |  |  |
| 20 | BG_GLEAN_10022504 | 14723300 | 14723737 |  |  |  |  |  |
| 21 | BG_GLEAN_10022508 | 14767873 | 14768121 |  |  |  |  |  |
| 22 | BG_GLEAN_10022531 | 15058961 | 15059329 |  |  |  |  |  |
| 23 | BG_GLEAN_10022553 | 15346449 | 15347135 |  |  |  |  |  |
| 24 | BG_GLEAN_10022555 | 15349247 | 15349756 |  |  |  |  |  |
| 25 | BG_GLEAN_10022720 | 17357350 | 17357901 | Transposase, MuDR, plant |  | transposase | [78] | DNA-binding |
| 26 | BG_GLEAN_10022734 | 17634365 | 17635519 | MuDR family transposase | binding |  |  |  |
| 27 | BG_GLEAN_10022402 | 13576671 | 13577819 | Zinc finger, CCHC-type | binding | DNA-binding protein | [79] |  |
| 28 | BG_GLEAN_10022652 | 16477031 | 16477843 |  |  |  |  |  |
| 29 | BG_GLEAN_10022770 | 18248778 | 18250028 |  |  |  |  |  |
| 30 | BG_GLEAN_10022515 | 14929579 | 14930304 | gag/pol protein |  | intracellular transport | [80] | Intracellular transport |
| 31 | BG_GLEAN_10022727 | 17456571 | 17458331 | Clathrin adaptor complex small chain family protein | intracellular protein transport  AP-3 adaptor complex |  | [81] |  |
| 32 | BG_GLEAN_10022776 | 18292930 | 18294105 | RNA-directed DNA polymerase (reverse transcriptase)-related family protein |  |  |  |  |
| 33 | BG_GLEAN_10022777 | 18295459 | 18295665 | DNAse I-like superfamily protein |  | catalysis | [82] | Catalysisenzyme |
| 34 | BG_GLEAN_10022276 | 12078272 | 12083573 | pyridoxal phosphate-dependent enzyme | metabolic process/pyridoxal phosphate binding/Cys or Met metabolism | oxidative biocatalysis | [83] |  |
